# Overlapping brain correlates of superior cognition among children at genetic risk for Alzheimer’s disease and/or major depressive disorder

**DOI:** 10.1038/s41598-023-28057-6

**Published:** 2023-01-18

**Authors:** Raluca Petrican, Amy L. Paine, Valentina Escott-Price, Katherine H. Shelton

**Affiliations:** 1 Institute of Population Health, Department of Psychology, University of Liverpool, Bedford Street South, Liverpool, L69 7ZA UK; 2 School of Psychology, Cardiff University, 70 Park Place, Cardiff, CF10 3AT UK; 3 Division of Neuroscience and Mental Health, School of Medicine, Cardiff University, Maindy Road, Cardiff, CF24 4HQ UK

**Keywords:** Genetics, Neuroscience, Psychology, Biomarkers, Neurology

## Abstract

Early life adversity (ELA) tends to accelerate neurobiological ageing, which, in turn, is thought to heighten vulnerability to both major depressive disorder (MDD) and Alzheimer’s disease (AD). The two conditions are putatively related, with MDD representing either a risk factor or early symptom of AD. Given the substantial environmental susceptibility of both disorders, timely identification of their neurocognitive markers could facilitate interventions to prevent clinical onset. To this end, we analysed multimodal data from the Adolescent Brain and Cognitive Development study (ages 9–10 years). To disentangle genetic from correlated genetic-environmental influences, while also probing gene-adversity interactions, we compared adoptees, a group generally exposed to substantial ELA, with children raised by their biological families via genetic risk scores (GRS) from genome-wide association studies. AD and MDD GRSs predicted overlapping and widespread neurodevelopmental alterations associated with superior fluid cognition. Specifically, among adoptees only, greater AD GRS were related to accelerated structural maturation (i.e., cortical thinning) and higher MDD GRS were linked to delayed functional neurodevelopment, as reflected in compensatory brain activation on an inhibitory control task. Our study identifies compensatory mechanisms linked to MDD risk and highlights the potential cognitive benefits of accelerated maturation linked to AD vulnerability in late childhood.

## Introduction

Early life adversity (ELA; e.g., poverty, maltreatment or neglect by a caregiver) is likely to require significant adaptation and can derail neurodevelopmental trajectories because the molecular brakes designed to facilitate normative maturational changes preserve instead the nocive consequences of ELA^1–3^. Systemic low-grade inflammation is a key mechanism through which ELA hinders optimal brain function by accelerating cellular senescence (e.g., DNA methylation for stress-relevant [serotonergic, glucocorticoid signalling] genes), and thus increases longer-term psychiatric and neurodegenerative risk^4–7^.

Among the neuropathologies typified by systemic inflammation and accelerated cellular ageing, Major Depressive Disorder (MDD) and Alzheimer’s Disease (AD) figure prominently as leading causes of disability worldwide^8–13^. Although only modestly genetically related, the two conditions are robustly linked to prior stress exposure, with disrupted synaptic transmission in the prefrontal cortex (PFC) being the alleged substrate of the cognitive control deficits that typify the clinical stage of both AD and MDD^5,14–19^. Recent literature suggests that MDD may be a risk factor or even an early symptom of AD^20^, which could help shed some light on the more subtle cellular changes which unfold decades before the clinical onset of AD^21^. Since many of the genetic factors linked to AD are under substantial environmental modulation^22^, characterisation of their early life neurocognitive correlates, including those shared with MDD and those susceptible to ELA exposure, could facilitate timely detection and identify avenues for intervention to decrease the risk for progression to dementia in older adulthood.

To our knowledge, most research on the brain correlates of AD and/or MDD risk has examined individuals raised by their birth families. These investigations cannot separate genetic from correlated ongoing non-genetic contributions to the observed phenotypes, as genetically vulnerable parents may create familial contexts that could either exacerbate (e.g., through reduced cognitive stimulation) or attenuate (via compensatory behaviours) their offspring’s risk for MDD/AD. Hence, any neurodevelopmental deviations in children at risk for AD/MDD who are raised by their birth families reflect both their own genetic vulnerability and their adjustment to the environment created by parents who may share their vulnerabilities.

To address the confounding effect of genetic effects and rearing environment, we characterised the neurocognitive correlates of genetic vulnerability to MDD/AD in late childhood (9–10 years) by comparing the profiles of adoptees and non-adoptees (i.e., children raised by their biological parents) who participated in the Adolescent Brain and Cognitive Development (ABCD) study. Inclusion of the adoptee group allowed us to (1) separate genetic from correlated gene-environment contributions to brain development, and (2) characterise the neurocognitive correlates of AD/MDD risk in a group exposed to environmental conditions thought to precipitate the onset of both disorders. Indeed, there is considerable evidence that adoptees tend to experience substantial ELA, as attested by both European and North American research^23–28^. For instance, according to current US adoption statistics^29^, of non step-parent adoptions (such as the majority of those herein investigated), approximately 60% involve children who have spent time in foster care, an experience usually preceded by substantial ELA exposure^24,26,30^. ELA has been linked to accelerated neurobiological maturation in childhood and adolescence^31^. While theorised to be an adaptative response to adverse rearing circumstances, which may optimise coping in the short-term^32^, accelerated maturation is likely to prevent fine-tuning of the slower developing brain circuits relevant to cognitive control, thereby increasing longer-term psychological vulnerability^33,34^, including risk for AD and MDD, respectively^19,20,35^.

Genetic risk scores (GRS) derived from genome-wide association studies (GWAS) quantified genetic liability to MDD and sporadic AD, respectively^36–38^. Two AD GRSs, one including only the Apolipoprotein E (APOE) region (i.e., APOE AD GRS) and a second excluding the APOE region (no-APOE AD GRS), were computed considering evidence that the two forecast distinguishable trajectories of neurocognitive impairments and differential susceptibility to environmental factors^22,39,40^. Specifically, APOE-based risk is associated with deviations in normative brain maturation from infancy onwards and predicts primarily memory-related deficits, stemming from progressive (medial) temporal and posterior parietal atrophy^22,41^. Complementarily, no-APOE-based risk for AD foreshadows a developmental trajectory of relatively greater deficits in cognitive control, language, and visuospatial processing, arising from a much larger progressive pattern of neurodegeneration which encompasses temporal, frontal, and parietal lobe structures^22^.

Cognitive control abilities, quantified with a so-called fluid cognition battery, constituted our core mental marker of MDD/AD risk due to their direct relevance to both pathologies, as well as their reported impact on lifespan neurogenetic and cardiovascular trajectories^19,42^. Because AD and MDD are typified by accelerated brain ageing^10^, developmental timing, estimated relative to other participants of the same chronological age, was examined as a brain marker for both. Neurodevelopmental timing was quantified with both structural and functional indices considering evidence that ELA impacts them differently^43^.

Cortical thickness was our index of structural neurodevelopmental timing due to its well-defined maturational trajectory, its liability to genetic control, as well as its susceptibility to ELA^44–47^. Functional neurodevelopmental timing was inferred from mean levels (BOLD_M_) and variability (BOLD_SV_) in blood oxygenation level dependent (BOLD) functional magnetic resonance imaging (fMRI) responses on a task probing inhibitory (attentional) control. Inhibitory control nears maturation in late childhood and is the cornerstone of optimal mental performance^48^. In contrast, difficulties with inhibitory control, which tend to be observed among adoptees^49^, constitute a transdiagnostic contributor to psychopathology^50^. Our interest in BOLD_M_ stemmed from its relevance to functional maturation, since similar behavioural performance on identical tasks is linked to greater BOLD_M_ in children relative to adults^51^. Complementarily, our focus on BOLD_SV_ was prompted by its susceptibility to developmental changes and its transdiagnostic involvement in psychopathology and remission following treatment^52–55^. To account for mental state-specific effects and their differential relevance to MDD/AD risk^56^, BOLD_SV_ was assessed during the externally oriented inhibitory control task and during wakeful rest. The latter tends to trigger an internally oriented attentional focus, which is key to MDD, while also evoking mind wandering episodes, which reportedly capture AD-linked deficits in spontaneous cognition^57–59^. Greater BOLD_SV_ during rest, particularly for brain regions implicated in externally oriented processing^56^, and reduced BOLD_SV,_ operationalised as reduced variability in task-evoked activation during inhibitory control performance, were regarded as indices of greater functional maturation.

Parenting can either accentuate or dampen the impact of ELA and/or genetic vulnerability to psychopathology^23,25,60–62^. Indeed, affective enrichment in childhood, including responsive parenting, can lessen the sequelae of earlier ELA exposure, and adoptive parental warmth reportedly fosters superior cognitive functioning, including inhibitory control^63–69^. Consequently, we investigated whether children’s perceptions of parental warmth would moderate the impact of AD and/or MDD genetic risk on neurodevelopmental timing and fluid cognitive abilities.

**Figure 1:**
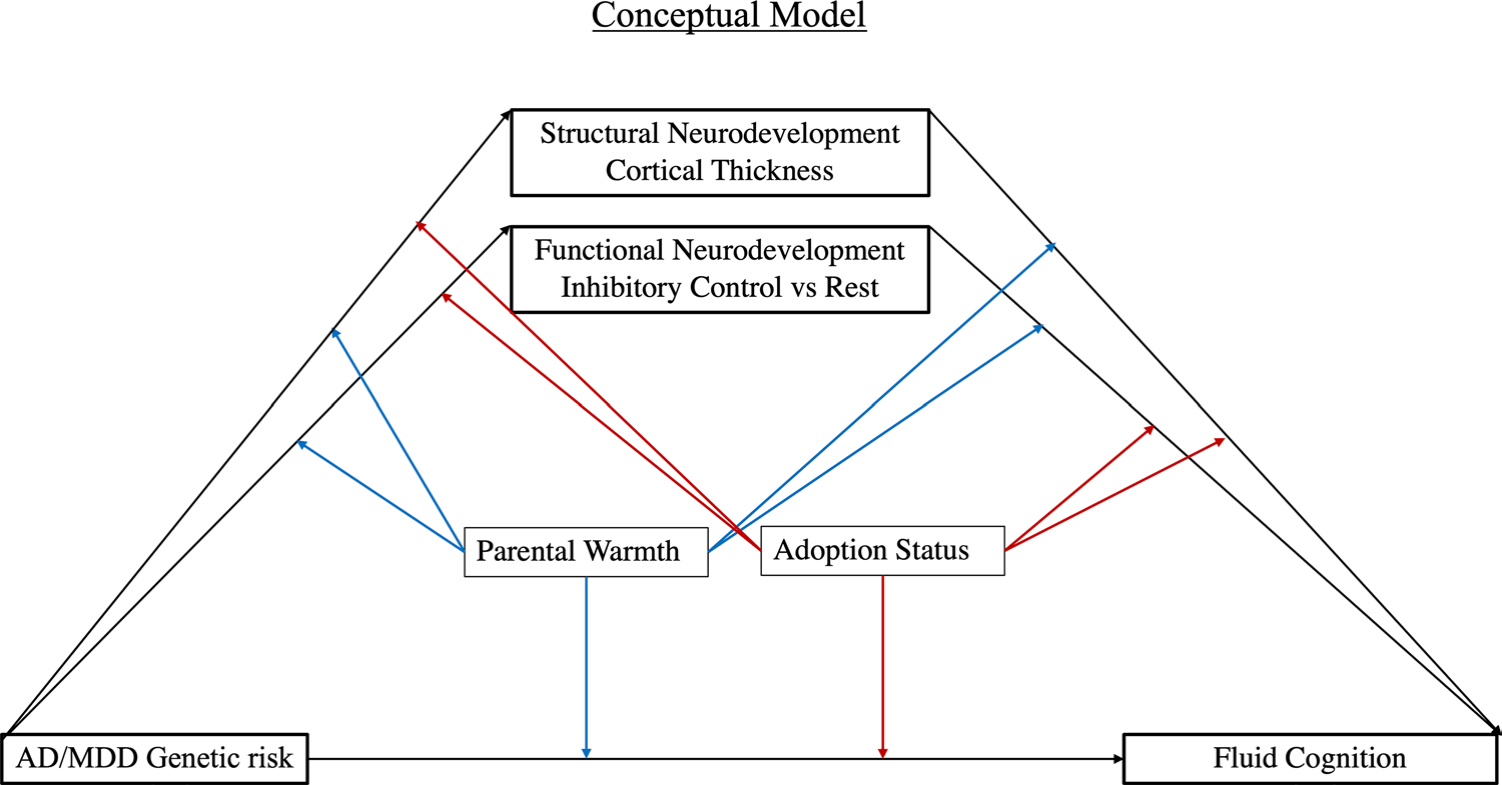
Outline of our conceptual model. Genetic risk for AD and/or MDD was predicted to accelerate structural and functional neurodevelopment, as reflected in patterns of cortical thickness and BOLD fMRI signal values during rest and performance of an inhibitory control task, respectively (see “Method” for details). Accelerated structural and/or functional neurodevelopment was expected to be linked to poorer fluid cognition scores. Adoption status and parental warmth were tested as potential moderators of the genetic risk-neurodevelopment-fluid cognition inter-relationships. *AD* Alzheimer’s Disease, *MDD* Major Depressive Disorder.

In sum, because accelerated brain ageing typifies both AD and MDD^10^, we tested whether genetic loading for either disorder is linked to earlier structural and/or functional neurodevelopment in late childhood. The hypothesised accelerated maturation associated with AD/MDD risk was expected to hinder the fine-tuning of the slower developing neurocognitive circuits relevant to inhibitory control^33,34^, and, thus, in turn, predict poorer fluid cognition (see Fig. 1 for a representation of our model). The inclusion of adoptees allowed us to disentangle gene-environment correlations and characterise “purer” neurocognitive correlates of AD/MDD genetic risk in a group in which their likely substantial ELA exposure may have amplified the impact of genes on vulnerability to accelerated neurobiological ageing^31^ and stress-linked pathologies, such as AD and MDD^19,20,35^. Secondly, we probed whether perceptions of high parental warmth would attenuate the adverse effect of MDD/AD risk on neurodevelopment and fluid cognition skills.

## Results

### Partial least squares (PLS) results

#### PLS 1: neurodevelopmental patterns differentiate between genetic risk for AD *vs.* MDD

**Figure 2:**
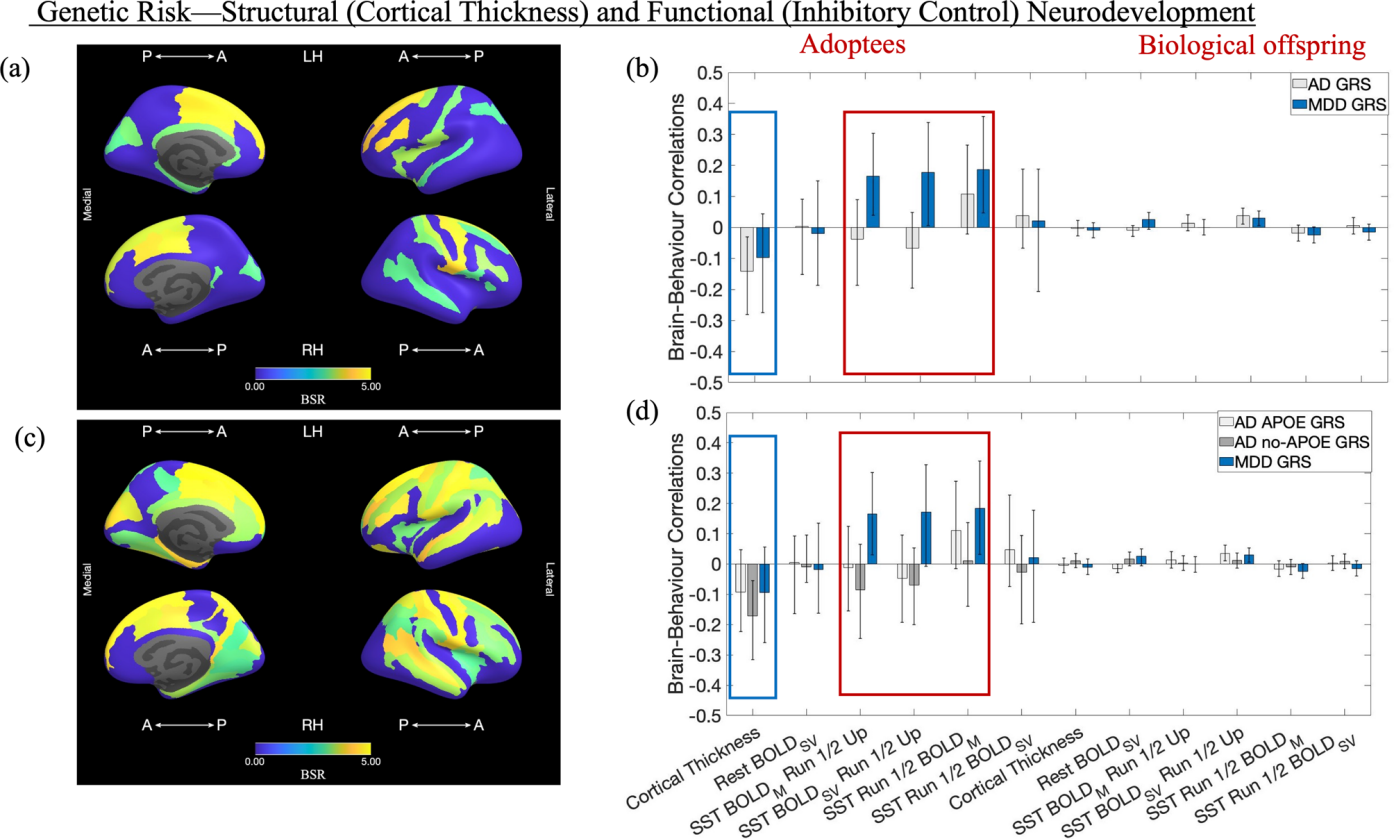
Results of the behavioural-PLS analyses. Panels (**b**) and (**d**) show the correlations between the LV brain scores and the GRSs. Error bars are the 95% CIs from the bootstrap procedure. CIs that do not include zero reflect robust correlations between the respective GRS and the brain score in a given condition (i.e., data type) across all participants. Panels (**a**) and (**c**) depict the Destrieux ROIs with robust loadings on the LVs in panels (**b**) and (**d**), respectively, and visualized with the Freesurfer Surface (https://chrisadamsonmcri.github.io/freesurfer_statsurf_display). In the brain figures in panels (**a**) and (**c**), absolute BSR values lower than 3 have been set to zero. Rest BOLD_SV_ = amplitude index of resting state low frequency fluctuations in BOLD signal. SST BOLD_M_ Run 1/2 Up = difference between the GLM-derived run 2 and run 1 betas, based on the Correct Stop > Correct Go contrast. SST BOLD_SV_ Run 1/2 Up = difference between the GLM-derived standard errors associated with the run 2 and run 1 beta, respectively, based on the Correct Stop > Correct Go contrast. SST Run 1/2 BOLD_M_ = average of the GLM-derived run 2 and run 1 betas, based on the Correct Stop > Correct Go contrast. SST Run 1/2 BOLD_SV_ = average of the GLM-derived standard errors associated with the run 2 and run 1 beta, respectively, based on the Correct Stop > Correct Go contrast. *LV* latent variable, *CI* confidence interval, *LH* left hemisphere, *RH* right hemisphere, *AD* Alzheimer’s Disease, *APOE* Apolipoprotein E, *MDD* Major Depressive Disorder, *GRS* genetic risk score, *SST* Stop-Signal Task, *GLM* general linear model, *BOLD* blood oxygenation level dependent.

The first PLS analysis revealed one significant latent variable (LV) (*p* = 0.0004), accounting for 53.93% of the GRS-brain data covariance. This LV distinguished AD from MDD GRS-linked brain markers (Fig. 2b) and was most strongly expressed in frontal, insular, parieto-occipital, and temporal areas (see Fig. 2a). Higher AD GRS was associated with greater cortical thinning among adoptees but increasing run 1 to run 2 BOLD_SV_ on the inhibitory control task (i.e., the stop-signal task [SST]) among non-adoptees. Complementarily, in both groups, higher MDD GRS was linked to increasing run 1 to run 2 BOLD_SV_ on the SST, as well as increasing cross-run BOLD_M_ and greater overall BOLD_M_ among adoptees only.

#### PLS 2: APOE- vs. no-APOE-based genetic vulnerability to AD is linked to distinct neurodevelopmental markers

The second PLS analysis identified a sole significant LV (*p* = 0.0002), which accounted for 47.18% of the covariance in the GRS-brain data and differentiated among brain markers of genetic risk for MDD, as well as APOE- vs no-APOE-linked vulnerability to AD (Fig. 2d). The associated brain LV was most strongly expressed in frontal, parietal, superior temporal, mid-posterior cingulate and parahippocampal gyri, as well as in occipito-temporal areas (Fig. 2c). The neural markers of MDD GRS comprised the same functional data types as those observed in the first PLS analysis. However, distinguishable brain correlates were observed for the two AD GRSs. Specifically, higher APOE GRS predicted increasing run 1 to run 2 BOLD_SV_ on the SST among non-adoptees, whereas higher no-APOE GRS predicted greater cortical thinning among adoptees.

### Supplemental tests

PLS analyses including only White children confirmed that racial differences in genetic architecture and risk loci^70–72^ did not impact our reported results (see Supplemental Materials).

### Moderated mediation analyses: genetic risk effects on brain and cognition among adoptees *vs.* non-adoptees

To investigate whether the PLS-identified neural correlates of AD and MDD risk mediate the genetic vulnerability-fluid cognition links, and whether any observed associations differ by adoption status, we conducted a series of moderated mediation analyses in which adoption status and parental warmth were entered as moderators of the GRS-brain, brain-fluid cognition and GRS-fluid cognition links, whereas crystallised cognition was introduced as a covariate. The predictors, mediators, moderator (parental warmth only) and outcomes of these analyses were residualised by the variables detailed in section “Residualisation for confounding variables” below and standardised separately within the adoptee and non-adoptee group, respectively. Our moderated mediational analyses focused on the GRS-brain associations identified by PLS to be significant in the adoptee group. However, in supplemental analyses, we verified that increasing run 1 to run 2 BOLD_SV_ on the SST did not mediate the link between the full or APOE-based AD GRS and fluid cognition (see Fig. 2b,d).

#### Composite AD GRS: cortical thickness as a mediator

**Figure 3:**
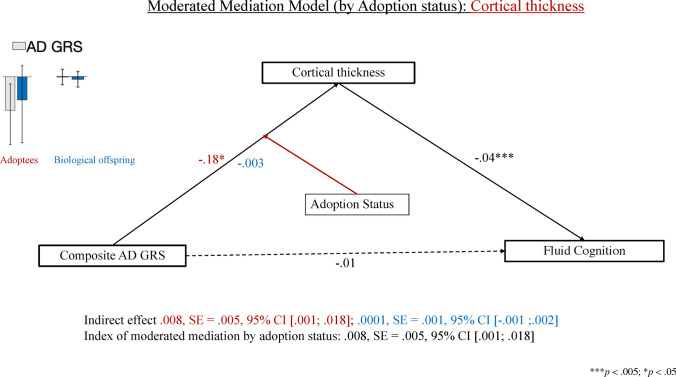
Results of the moderated mediation analysis comparing the effect of the composite AD GRS on fluid cognition via cortical thickness among adoptees and non-adoptees. *AD* Alzheimer’s Disease, *GRS* genetic risk score. Coefficients in red font describe the adoptees, those in blue font, the biological offspring, whereas those in black font apply to the full sample.

Our analysis revealed that adoption status (but not parental warmth) partially moderated the indirect effect of AD GRS on fluid cognition via cortical thickness (see Fig. 3). Specifically, among adoptees (but not non-adoptees), higher AD GRS was related to greater cortical thinning (than expected by chronological age), suggestive of accelerated structural neurodevelopment, which, in turn, predicted superior fluid cognition scores among both adoptees and non-adoptees.

##### Explained variance in fluid cognition scores

Follow-up correlation-based analyses revealed that the composite AD GRS explained 0.4% (adoptees) and 0.01% (non-adoptees), respectively, whereas cortical thickness explained 0.2% (no statistically significant group difference as per the moderated mediation results above) of the variance in fluid cognition scores.

#### No-APOE AD GRS: cortical thickness as a mediator

**Figure 4:**
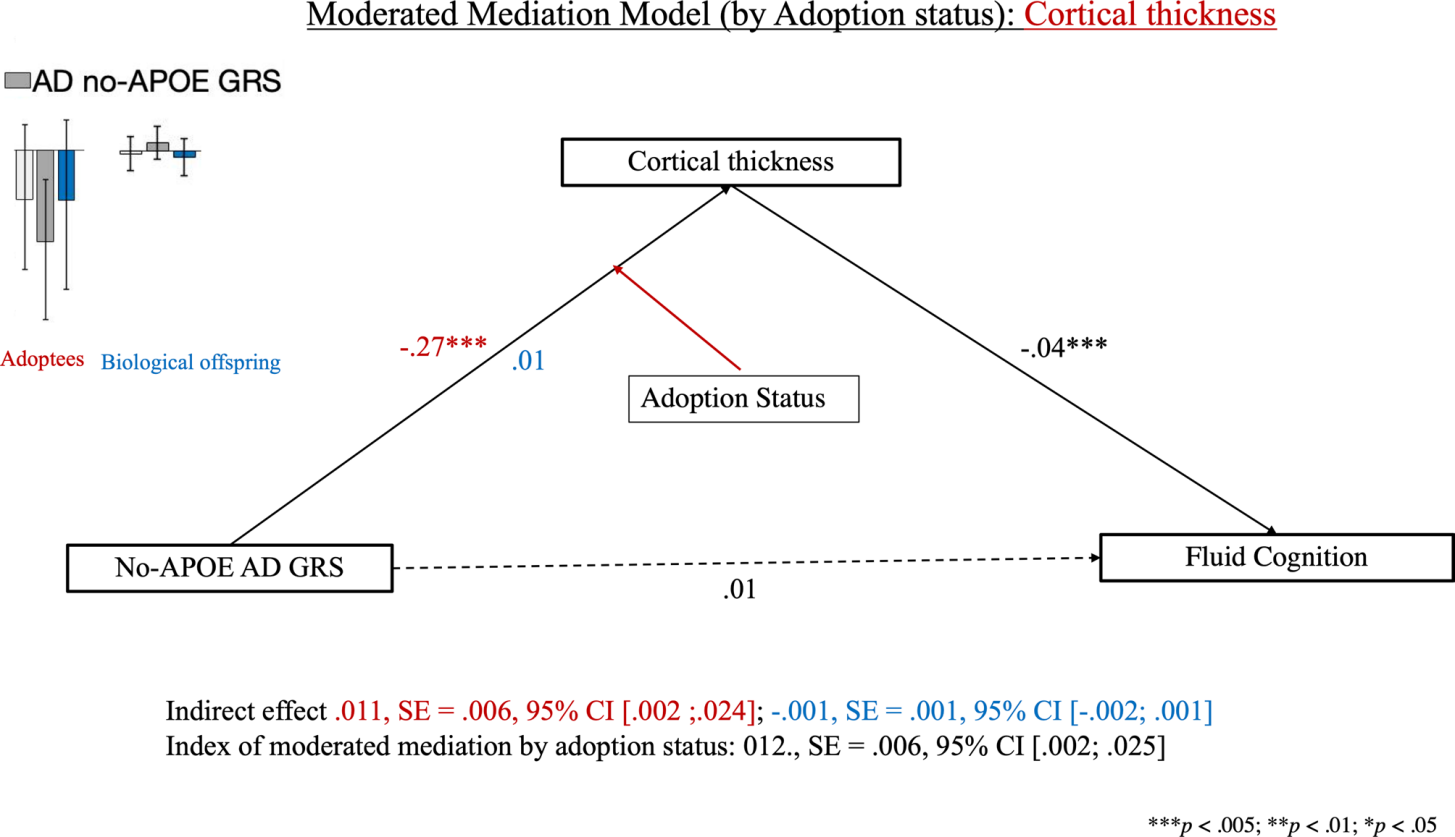
Results of the moderated mediation analysis comparing the effect of the no-APOE AD GRS on fluid cognition via cortical thickness among adoptees and non-adoptees. *AD* Alzheimer’s Disease, *APOE* Apolipoprotein E, *GRS* genetic risk score. Coefficients in red font describe the adoptees, those in blue font, the biological offspring, whereas those in black font apply to the full sample.

Adoption status, but not parental warmth, partially moderated the link between no-APOE AD risk and fluid cognition via cortical thickness (see Fig. 4). Thus, replicating the results obtained with the composite AD GRS, we found that among adoptees (but not non-adoptees), higher no-APOE-based AD GRS was linked to greater cortical thinning, which, in turn, predicted higher fluid cognition scores among both adoptees and non-adoptees.

##### Explained variance in fluid cognition scores

Follow-up correlation-based analyses revealed that the no-APOE AD GRS explained 0.2% (adoptees) and 0.02% (non-adoptees), respectively, whereas cortical thickness explained 0.2% (no statistically significant group difference as per the moderated mediation results above) of the variance in fluid cognition scores.

#### MDD GRS: SST BOLD_M_ (average and cross-run increase) and BOLD_SV_ (cross-run increase) as parallel mediators

**Figure 5:**
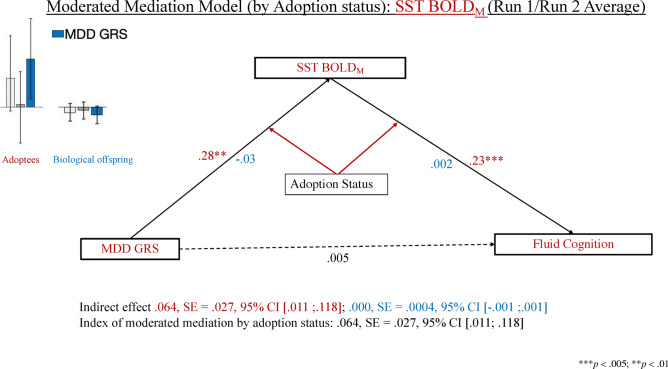
Results of the moderated mediation analysis comparing the effect of the MDD GRS on fluid cognition via average SST BOLD activation among adoptees and non-adoptees. *MDD* Major Depressive Disorder, *GRS* genetic risk score, *SST* Stop-Signal Task, *BOLD* blood oxygenation level dependent. Coefficients in red font describe the adoptees, those in blue font, the biological offspring, whereas those in black font apply to the full sample.

We observed a moderated mediation effect by adoption status (but not parental warmth) indicating that the indirect effect of MDD GRS on fluid cognition via SST BOLD_M_ was significant among adoptees but not among non-adoptees (see Fig. 5). Specifically, among adoptees only, higher MDD GRS values predicted higher SST BOLD_M_, which, in turn, was associated with superior fluid cognition scores.

##### Explained variance in fluid cognition scores

Follow-up correlation-based analyses revealed that the MDD GRS explained 1% (adoptees) and 0.01% (non-adoptees), respectively, whereas SST BOLD_M_ explained 8.4% (adoptees) and 0.0004% (non-adoptees), respectively, of the variance in fluid cognition scores.

## Discussion

The present study provides novel evidence of late childhood neurodevelopmental alterations which are related to genetic vulnerability for AD and MDD, respectively. Contrary to our hypotheses, the observed maturational deviations were linked to superior, rather than poorer, fluid cognitive performance and emerged only among adoptees, a group likely to have experienced substantial ELA^26,30^. As such, our findings reaffirm the role of stress exposure in both MDD and AD^19,20,35^, as well as the importance of disentangling correlated gene-environment influences to better characterise the intergenerational transmission of adaptive and pathological functional profiles.

In line with the proposed role of synaptic dysregulation in the PFC as a key contributor to both AD- and MDD-related pathologies^19^, we report a link between genetic risk for either disorder and developmental alterations in this region. Reinforcing the key role of cognitive control in both pathologies^19^, we show that the broader maturational brain profiles linked to AD and MDD vulnerability overlap with regions robustly implicated in intentional decision-making, working memory performance and lifespan fluctuations in cognitive flexibility^73,74^. Furthermore, complementing prior investigations on the relevance of APOE-based AD risk to brain development from infancy onwards^41^, we demonstrate that no-APOE-based genetic risk accounts for the observed overlap in the neurodevelopmental alterations related to MDD and AD GRSs in late childhood, at least among children not raised by their biological families. As the strongest effects emerged in frontal and parietal regions and were related to performance on a fluid cognition battery, our findings corroborate the documented neurodegenerative and mental (i.e., memory- vs cognitive control-related) profile that distinguishes no-APOE from APOE-based risk^22^.

It is important to note that the neural patterns linked to AD risk reflected structural developmental deviations, whereas those associated with MDD risk indicated functional alterations. The stronger heritability of structural (relative to functional) brain indices^75^ raises the possibility that our AD-related findings may reflect genetic influences to a greater extent than our MDD-linked results. For AD, this interpretation is bolstered by the fact that the GRS-accelerated structural neurodevelopment link replicates the genetically influenced brain print of this pathology^10^, implying that accelerated brain maturation linked to AD vulnerability may yield some cognitive benefits, albeit modest, in late childhood.

As for MDD, the GRS-brain associations were in the opposite direction to those generally documented for this disorder in adulthood^8–10,12^. One possibility is that the brain print of MDD varies by age, with accelerated/decelerated development/ageing both likely to typify this disorder, but at different life stages and yielding distinct functional outcomes. An alternative interpretation, based on the specificity of the GRS-brain-cognition relationships to the adoptees, is that the MDD-related findings reflect compensatory mechanisms arising from an interaction between genetic vulnerability and prior ELA exposure, which tends to be higher in this group^26,30^. Although adverse life experiences were predicted to accentuate vulnerability to AD and MDD by accelerating brain ageing and interfering with the fine tuning of brain circuits involved in cognitive control^19,20,33,34^, this may not always be the case. Indeed, there is suggestive evidence that exposure to harsh and unpredictable circumstances, as it plausibly applies to adoptees^26,30^, can also foster the development of certain cognitive control components^76^, some of which were captured by the ABCD fluid cognition battery. In this context, the MDD-associated GRS-brain-cognition relationships may be accommodated within the framework of differential susceptibility^77^, as genetic risk for MDD among adoptees may accentuate responsiveness not only to the adverse, but also to the beneficial (e.g., mental flexibility-promoting^76^) aspects of their early life environments.

Although our main analyses showed AD GRS-linked structural and MDD GRS-related functional developmental alterations, our supplemental tests using more lenient significance thresholds for the GRS-contributing single nucleotide polymorphisms (SNPs) identified accelerated cortical thinning as a common feature of both AD and MDD genetic vulnerability, thereby replicating the brain print of both disorders^10^. Taken together, our results highlight the interplay between genetic and environmental (i.e., adversity-related) contributions to MDD-related developmental deviations in function versus structure, some of which appear to play a compensatory role.

Among children raised by their birth parents, the only significant GRS-brain association suggested that greater vulnerability to AD and/or MDD is linked to decreased robustness in neural activity on the inhibitory control task. Although the underpinnings of this effect need to be probed in more depth, it seems plausible that decreased attentional focus may, at least, partially explain our finding, in line with the broadly posited association of poorer cognitive control with both AD and MDD^19^. It is worth noting that, consonant with prior rodent findings^78^, among non-adoptees, an overlap in neurodevelopmental alterations was detected for MDD and APOE-, rather than no-APOE-, based AD risk. These results suggest that overlapping maturational deviations linked to MDD and no-APOE-based AD vulnerability may indicate primarily direct genetic effects, while shared neural alterations associated with MDD and APOE-based AD liability may stem from correlated gene-environment influences, reflecting both the direct and indirect impact of genes.

Contrary to our predictions, parental warmth did not moderate the association between genetic risk and neurocognitive development among either adoptees or non-adoptees. However, in our sample, children’s ratings of parental warmth were very high and showed relatively little variability. Consequently, more in-depth longitudinal investigations with measures spanning cellular to functional systems levels are warranted to elucidate the buffering role of parent–child relationship quality, including parental warmth and responsiveness^79^. Mounting evidence testifies to the profound impact of parenting on child development. For instance, child maltreatment is linked to epigenetic changes in oxytocin function, which, in turn, trigger structural and functional brain alterations relevant to reward and external attention processes^80^. Conversely, both rodent and human (adoption) studies indicate that child and adolescent exposure to enriched and emotionally responsive environments can reverse the sequelae of prior ELAs (e.g., restored adult hippocampal neurogenesis, reductions in stress reactivity and biological ageing^64,65,67,81^). Consequently, the neurobiological mechanisms through which enriched social environments may compensate for the damage inflicted by earlier exposure to harsher milieus, as well as the potential age-specificity of any detected pathways would be certainly worth exploring in the future.

Our research paves the way for several lines of inquiry. First, use of recently discovered genetic risk loci and more liberal significance thresholds for the GRS-contributing SNPs^82–84^ could help elucidate the clinical symptoms and underlying neural circuitry linked to accelerated brain ageing as a function of MDD and/or APOE vs no-APOE AD risk (e.g., anhedonia^85,86^; sleep disturbances^87^). Such investigations could also characterise the molecular pathways through which APOE variants (APOE 2 vs APOE 4) may either protect against or increase vulnerability to ageing-related cognitive decline^88^, thereby optimising screening and intervention for at-risk individuals.

Second, the cellular substrates of gene-perinatal environment interactions in neurodegeneration and psychopathology, particularly those relevant to fluid cognition, warrant further investigation (e.g., E/I imbalance, neurotransmitter-specific alterations^3,89^). Such inquiries are well-justified by evidence that pre-/perinatal stressors (e.g., malnutrition, maternal trauma, inflammation, psychopathology, substance use) interact with the offspring’s genetic profile to shape their lifespan development, including biological ageing rate^90–92^.

Third, given the domain-specificity of inhibitory control^93^, the differential neurocognitive alterations on general *vs.* emotional context-specific tasks, linked to AD *vs.* MDD risk, are worth probing. Fourth, the relatively small sample of adoptees did not provide us with sufficient statistical power to test for sex differences in any of the documented gene-brain-cognition relationships. Such investigations are worth pursuing because there are sex differences in the prevalence of both AD and MDD^94,95^, metabolic brain senescence, age-, APOE-4 status-related AD risk, as well as in the neuro(epi)genetic profiles and intergenerational transmission patterns of psychopathology^88,96–98^.

Fifth, the well-documented neurogenetic and adverse life outcome overlap in psychiatric and degenerative brain disorders highlight the importance of disentangling the alterations in neurocognitive development specific to AD and MDD, respectively, from those associated with global vulnerability to psychopathology^10,99–101^. For instance, in our study, the brain areas linked to AD/MDD risk overlap with those of a recently characterised “vulnerability network” implicated in global psychiatric risk, substance use and educational attainment^102^. Thus, although some regionally constrained effects may exist, disorder-specific pathological profiles (including MDD/AD) may reflect not variability in the topography of the affected brain network, but rather fluctuations in the precise combination of cross-modality (structure/function) deficits.

Sixth, there is a need for systematic investigations of a wider range of ELAs (e.g., poor sleep quality, air pollution, urbanicity) and of likely buffers, be they dispositional/person-related (e.g., purpose in life, self-control, emotion regulation skills, educational attainment) or environmental/lifestyle-related (e.g., green space exposure, nutritional supplement use, aerobic engagement)^103–107^. Seventh, although we controlled for many demographic variables, lingering differences between adoptees and non-adoptees could have still impacted our reported findings. Our present results based on a relatively small adoptee sample need to be replicated and extended in studies using a combined twin/GRS approach with biological and adoptive families, including an in-depth demographic assessment, well-documented adoption route (e.g., foster care/international/step-family) and pre-adoptive history of adversity. Such investigations could elucidate direct *vs*. indirect genetic, as well as bidirectional parent–offspring effects on the AD- and MDD-relevant phenotypes^108,109^.

In sum, we identified overlapping neurodevelopmental mechanisms linked to superior fluid cognition among adoptees at genetic risk for AD and MDD, respectively. The AD-related structural profile replicated the accelerated brain ageing print of this disorder, while highlighting its unexpected, albeit modest, cognitive benefits in early life. The MDD-linked functional profile was the reverse of its neuropathological print and reflected compensatory mechanisms likely related to prior adversity exposure.

## Methods

### Participants

We used data pre-processed by the ABCD study team (ABCD data release 3.0) from 117 adoptees and 4382 non-adoptees, aged 9–10 years, who were biologically unrelated and provided high-quality data on all scrutinised measures (see Table 1 for detailed demographic information). The majority (72%) were confirmed non step-parent adoptions. In the remaining 28% of the cases, the mother was confirmed to be not biologically related to the child (i.e., adoptive), whereas information on the biological relatedness of the father to the child was missing.

**Table 1:** Demographic and genetic risk information for adoptees and non-adoptees.

Variable	Adoptees N = 117	Non-adoptees N = 4382
Age (years)	9.96 ± 0.60	9.94 ± 0.63
Sex (F/M)	59/58	2184/2198
Handedness (% mostly right-handed)	75%	82%
Youth race	White only (33%)	White only (73%)
	Black only (35%)	Black only (10%)
	Asian only (9%)	Asian only (1%)
	Other/mixed race (23%)	Other/mixed race (16%)
Participating parent race*	White only (73%)	White only (78%)
	Black only (14%)	Black only (10%)
	Asian only (3%)	Asian only (3%)
	Other/mixed race (10%)	Other/mixed race (9%)
Family income (USD)**	0 to 24,999 (7%)	0 to 24,999 (10%)
	25,000 to 49,999 (11%)	25,000 to 49,999 (13%)
	50,000 to 74,999 (15%)	50,000 to 74,999 (14%)
	75,000 to 99,999 (21%)	75,000 to 99,999 (16%)
	100,000 to 199,999 (36%)	100,000 to 199,999 (34%)
	200,000 + (10%)	200,000 + (14%)
Material deprivation	0.26 ± 0.83	0.37 ± 0.98
Participating parent education*	Graduate school (40%)	Graduate school (29%)
	Four-/three-year college (26%)	Four-/three-year college (32%)
	One-/two-year college (22%)	One-/two-year college (28%)
	Highschool (12%)	Highschool (11%)
Crystallised cognition	85.70 ± 6.32	87.94 ± 6.62
APOE AD PRS	0.023 ± 0.030	0.028 ± 0.033
No-APOE AD PRS	0.007 ± 0.013	0.003 ± 0.012
MDD PRS	0.000 ± 0.003	0.000 ± 0.003
AD PRS	0.016 ± 0.018	0.018 ± 0.020

*GRS* genetic risk score, *AD* Alzheimer’s Disease, *MDD* Major Depressive Disorder. *Due to data availability, we present racial and educational information for the participating parent who tended to be the mother in both adoptive and birth families (91% for both). **Only 103 adoptive families reported income.

### Out-of-scanner measures

Scores on all the measures were released by the ABCD team and are described in detail in^110^ (see Supplementary Materials 1.2–1.4). Fluid and crystallised cognition were assessed with the National Institutes of Health (NIH) Toolbox. Unadjusted (rather than age-/sex-adjusted) scores were used for both to avoid interference with our numerous confounding variables, which included age and sex (see section on confound residualisation below). Fluid cognition scores reflected average performance on inhibitory control, cognitive flexibility, working memory, processing speed and episodic memory tests. Crystallised cognition scores indicated average performance on receptive vocabulary and oral reading tasks.

Children’s perceptions of parental warmth were gauged with the Acceptance subscale from the Child Report of Behavior Inventory. Concurrent adversity was quantified through parental responses on a measure of unmet material needs, as well as parent and child responses on two measures assessing family conflict and neighbourhood crime.

### Neuroimaging data

We used tabulated structural (i.e., cortical thickness) and functional magnetic resonance imaging (fMRI: resting state and task-related) data pre-processed by the ABCD team and mapped onto the 148 regions-of-interest (ROIs) in the Destrieux anatomical atlas (see Supplementary Materials 1.5–1.7). The task fMRI data had been collected during performance of a stop-signal task (SST) which measures the ability to inhibit an ongoing speeded motor response to a “Go” signal^110^. Our SST analyses focused on the beta and associated standard error (SEM) values derived from the Correct Stop > Correct Go contrast, as estimated with a general linear model (GLM) in the Analysis of Functional NeuroImages (AFNI^111^). There was no reason to control for behavioural performance because: (1) we only analysed correct trials, and (2) task difficulty was dynamically adjusted to maintain a set number of correct responses across participants^110^.

Two BOLD_M_ -related estimates were computed for each of the 148 Destrieux ROIs based on the Correct Stop > Correct Go contrast. The first was the difference in standardised GLM beta values between the second and the first run of the SST task. Lower values on this measure typified individuals who became more “brain-efficient” with practice (i.e., correct performance was linked to less neural activity on run 2 relative to run 1, cf.^112–114^). The second BOLD_M_ -related estimate was the average GLM beta value across the two runs of the SST task. Lower values on this measure characterised participants with overall greater neural efficiency, likely indicative of greater functional maturation (cf.^51^).

Based on the Correct Stop > Correct Go contrast, two BOLD_SV_-related indices were also estimated for each of the 148 Destrieux ROIs. Both were based on the SEM associated with the GLM beta coefficient for the Correct Stop > Correct Go contrast. The first BOLD_SV_-related index was computed as the difference between the run 2 and run 1 standardised SEM values. Lower values on this measure identified participants who showed greater stabilisation of the task-related response from run 1 to run 2 (relative to the sample mean). The second index was the average run 1 and run 2 SEM, with lower values typical of participants with a more consistent response to the task-relevant information. Resting state BOLD_SV_ was estimated as an amplitude index of low frequency fluctuations.

### Genetic risk scores (GRS)

MDD and AD (full, APOE [chromosome 19:44.4–46.5 Mb] and no-APOE) GRSs were each computed as the weighted sum of risk alleles, significant at GWAS level *p* ≤ 5 × 10^–8^. These were derived from the summary statistics of two large GWASs focused on each disorder^36,37^ (see Supplementary Materials 1.8). The absence of the relevant SNPs (rs7412 and rs429358) from the quality controlled ABCD genetic data prevented us from computing the APOE AD GRS as the sum of e4/e2 alleles^40^. Hence, as stated above, the APOE AD GRS was estimated as the weighted sum of risk alleles in the APOE region, thereby using the same procedure as for the other GRSs.

### Residualisation for confounding variables

To minimise bias in our multivariate brain-behaviour analyses^115^, only the non-imaging variables were residualised for the following confounders: sex, race (separate dummy-coded variables for “Black”, “Asian”, ‘Mixed Race” regressed simultaneously from the non-imaging variables to account for potential differences between these racial groups and White participants), handedness, serious medical problems, scanner site, material deprivation, family conflict, neighbourhood crime, age at adoption, average modality-specific motion per participant, and chronological age (in order to estimate accelerated/decelerated neurodevelopment relative to the other participants) (see Supplementary Materials 1.9). Due to data (un)availability, only the non-adoptee data were residualised for perinatal adversity, as indexed by a summary score released by the ABCD team and reflecting maternal prenatal care, maternal substance use during pregnancy, prenatal maternal health conditions, prematurity, birth complications and developmental milestones^116^. The adoptee and non-adoptee data were residualised separately.

### MRI and GRS data analysis

To characterise the relationship between MDD/AD risk and neurodevelopmental timing, we used partial least squares correlation (PLS^117^), a multivariate technique that can identify in a data-driven manner neural patterns (i.e., latent variables or LVs) related to different conditions (task PLS) and/or individual differences variables (behavioural PLS) (see Supplementary Materials 1.10.1). We conducted two behavioural PLS analyses featuring the MDD GRS (both analyses) and either the composite AD GRS (analysis 1) or the APOE- vs no-APOE AD GRSs (analysis 2) in the “behavioural” set. The brain matrix contained the coefficients corresponding to each brain data type, which was modelled as a separate condition (i.e., “cortical thickness”, “Rest BOLD_SV_” [= amplitude of resting state low frequency fluctuations in BOLD signal, as released by the ABCD team], “SST BOLD_M_ Run 1/2 Up” [= difference between the GLM-derived run 2 and run 1 betas, based on the Correct Stop > Correct Go contrast], “SST BOLD_SV_ Run 1/2 Up” [= difference between the GLM-derived SEMs associated with the run 2 and run 1 beta, respectively, based on the Correct Stop > Correct Go contrast], “SST Run 1/2 BOLD_M_” [= average of the GLM-derived run 2 and run 1 betas, based on the Correct Stop > Correct Go contrast, as released by the ABCD team], “SST Run 1/2 BOLD_SV_” [= average of the GLM-derived SEMs associated with the run 2 and run 1 beta, respectively, based on the Correct Stop > Correct Go contrast, as released by the ABCD team]). The adoptees and non-adoptees were modelled as separate groups. LV significance was determined with 5000 permutations, whereas the reliability of each ROI’s contribution to a particular LV was quantified based on the standard error estimates (SEs) from 1000 bootstraps^117,118^. A bootstrap ratio (BSR) (weight/SE) of 3 in absolute value (conceptually similar to an associated *p*-value < 0.003) was used as a robustness threshold for all ROIs (cf.^117,118^).

Three moderated mediation analyses using Hayes’ PROCESS 3.5 macro for the Statistical Package for the Social Sciences (SPSS^119^) probed whether MDD, composite AD and APOE vs no-APOE AD GRSs predicted distinct neurocognitive developmental patterns among adoptees versus non-adoptees (see Supplemental Materials 1.10.2). Mediation models were tested employing 95% CI (percentile bootstrap, 50,000 bootstraps) with a heteroscedasticity consistent standard error and covariance matrix estimator.

## Supplementary Information

Supplementary Information.

## Data Availability

The raw data are available at https://nda.nih.gov/abcd upon completion of the relevant data use agreements. The ABCD data repository grows and changes over time. The ABCD data used in this report came from Adolescent Brain Cognitive Development Study (ABCD)—Annual Release 3.0 #901. DOIs can be found at https://doi.org/10.15154/1519007.

## Code availability

We used existing code, as specified in the main text with links for free download.
