# Supplementary material for: Overlapping brain correlates of superior cognition among children at genetic risk for Alzheimer’s disease and/or major depressive disorder
Source: Sci Rep. 2023 Jan 18;13:984. doi: 10.1038/s41598-023-28057-6 (PMC9849214; doi:10.1038/s41598-023-28057-6)
Supplement: Supplementary file 1 — Supplementary Information. [file 41598_2023_28057_MOESM1_ESM.pdf]

Supplemental Materials

1. Methods

1.1 Participants

The sample included biological and adoptive parents and offspring who participated in the ongoing Adolescent Brain Cognitive Development (ABCD) study (for a detailed sample description, see [1]).

The present research uses data preprocessed by the ABCD study team and downloaded in December 2020 as part of the ABCD Study Curated Annual Release 3.0 (https://data-archive.nimh.nih.gov/abcd). Following the recommendations of the ABCD study team (as detailed in the "abcd_imgincl01" file included in the data release), 117 adoptees (59 female) and 4382 non-adoptees (2184 female) were selected on the basis of being biologically unrelated and having contributed high-quality data on all measures of interest. Participants were aged 9-10 years (adoptees: $M = 119.46$ months, $SD = 7.23$ months; non-adoptees: $M = 119.36$ months; $SD = 7.50$ months) and the majority were predominantly right-handed (N = 88 [adoptees]; N = 3575 [non-adoptees]). Age at adoption ranged from 0 to 10 years ($M = 2.27$ years, $SD = 2.50$ years). The majority (72%) were confirmed non-step-children adoptions (i.e., neither parent was biologically related to the offspring). In the remaining 28% of the cases, the mother was confirmed to be not biologically related to the child (i.e., adoptive), whereas information on the biological relatedness of the father to the child was missing. Demographic information on the sample is presented in Table 1.

1.2 Fluid Cognition

The fluid cognition battery is described in detail in [2].

1.2.1 Inhibitory Control

The National Institutes of Health (NIH) Toolbox Flanker Inhibitory Control and Attention Test gauged participants' ability to focus on a given target stimulus, while

inhibiting attention to stimuli flanking it. The task encompasses two types of trials, congruent (i.e., when the target stimulus is pointing in the same direction as the “flankers”) and incongruent (i.e., when the target stimulus is pointing in the opposite direction from the “flankers”). The participants' scores, included in the ABCD data release, were based on a combination of accuracy and reaction time (i.e., for participants with an accuracy rate of 80% or below, their total score equals their accuracy score, whereas for participants showing accuracy rates greater than 80%, their total score is an aggregate of their accuracy score, rescaled to the 0-5 range, and their median speed [reverse-scored reaction time] on the correct incongruent trials, also rescaled to the 0-5 range).

1.2.2 Cognitive Flexibility

The NIH Toolbox Dimensional Change Card Sort Test was used to assess cognitive flexibility. Participants are shown two target pictures, which vary along two dimensions (i.e., shape and color). They are then asked to match a series of bivalent pictures to the target pictures, first according to one dimension (e.g., color), then, after a number of trials, according to the second dimension (e.g., shape). The participants' scores, included in the ABCD data release, were based on a combination of accuracy and reaction time, as described above for inhibition.

1.2.3 Working Memory

The NIH Toolbox List Sorting Test indexed participants’ working memory capacity, specifically, their ability to encode and manipulate information. In the List Sorting task, participants are presented with pictures of foods or animals, each accompanied by a sound clip and written text that identify the respective item. List length varies from two to seven items. In the 1-List condition, participants have to arrange either food items or animals in size order from smallest to largest. In the 2-List condition, they are presented with food items as well as animals and are required to report the food items first in size order, followed by the

animals in size order. The outcome measure is the number of correctly recalled items (i.e., lists). For an item to be considered correct, all its constituents need to be reported in the correct size order.

1.2.4 Processing Speed

The NIH Toolbox Pattern Comparison Processing Speed Test gauged how quickly participants could decide whether two pictures, presented side by side, were identical or not. Processing speed is operationalized as the number of items correct in a 90-second period.

1.2.5 Episodic Memory

The NIH Toolbox Picture Sequence Memory Test assessed visual episodic memory. Participants are required to recall increasingly lengthier series of illustrated objects and activities presented in a specific order on a computer screen. Sequence length varies from 6 to 18 pictures. Participants are given credit for each pair of adjacent pictures correctly recalled up to the maximum value for each sequence, which is one less than sequence length.

1.3 Parental warmth/acceptance

An abbreviated five-item version of the Acceptance subscale from the Child Report of Behavior Inventory [3,4] assessed youth's perception of parental warmth and responsiveness with a 3-point Likert type scale (e.g., "Makes me feel better after talking over my worries with him/her"; "Smiles at me very often"). Because most participants did not rate their secondary caregiver, our analyses focused on evaluations of the primary caregiver's warmth (both Cronbach's alphas of .69, $M = 2.77$ [$SD = .30$] and $M = 2.80$ [$SD = .28$] for adoptive and biological parents respectively).

1.4 Control variables

The crystallised cognition battery is described in detailed in [2].

1.4.1 Crystallised Cognition (Language)

Vocabulary comprehension. The NIH Toolbox Picture Vocabulary Task, a variant of the Peabody Picture Vocabulary Test gauged participants' language skills and verbal intelligence. Following aural presentation of word stimuli, participants are shown four pictures from which they have to select the one that depicts the concept or object referenced by the word they had just heard. The outcome measure is the total number of auditorily presented words correctly matched to their corresponding picture.

Reading decoding. The NIH Toolbox Oral Reading Recognition Task assessed participants' exposure to language materials and their reading-relevant skills. The test requires individuals to pronounce single letters and words, which are scored by the tester as either correct or incorrect (based on accepted national norms). The outcome measure is the total number of correctly pronounced letters or words.

1.4.2 Material Deprivation

Financial deprivation was assessed with a 7-item scale to assess unmet material needs in the areas of housing, food and medical care in the 12 months preceding assessment (3, 4). Each item is scored as *1* or *0* (*yes/no*). Responses to all items were summed with higher scores indicating *experiences* of greater financial hardship (both Cronbach's alphas of .75, *M* = .26 [*SD* = .84] and *M* = .37 [*SD* = .99] for adoptive and biological parents, respectively).

1.4.3 Exposure to violence

Both measures of threat described below were completed independently by the parent and the youth as part of the wider PhenX Toolkit [3, 4].

Family conflict. A 9-item Family Conflict subscale gauged exposure to domestic violence. Each item is scored as *1* or *0* for *true*/*false*, with reverse coding of items that imply lack of conflict in the home (e.g., "We fight a lot in our family." versus "Family members rarely become openly angry."). Higher scores indicate a more conflictual family environment. Both parent (Cronbach's alphas of .68 [*M* = .30, *SD* = .23] and .67 [*M* = .27, *SD*

= .21] for adoptive and biological parents, respectively) and youth (Cronbach's alphas of .60 [*M* = .26, *SD* = .25] and .61 [*M* = .20, *SD* = .21] for adoptive and biological offspring, respectively) versions demonstrated acceptable reliability.

Perceived neighbourhood crime and safety. A 3-item Neighbourhood Safety/Crime Scale, using a five-point Likert Scale (*5 = strongly agree* to *1= strongly disagree*), gauged perceptions of threat related to the neighbourhood in which the respondent resides (i.e., areas within a 20-minute walk from the respondent's home). The youth completed only a 1-item version of the scale ("My neighbourhood is safe from crime"; *M* = 4.15 [*SD* = 1.00] and *M* = 4.13 [*SD* = 1.00] for adoptive and biological offspring, respectively), whereas the parent filled out the full 3-item version of the scale (Cronbach's alphas of .87 [*M* = 3.92, *SD* = .90] and .87 [*M* = 3.99, *SD* = .91] for adoptive and biological parents, respectively). Higher scores on this scale indicate greater neighbourhood safety.

1.4.4 Perinatal Adversity

An index of perinatal adversity was extracted through principal components analysis from caregiver responses on the Developmental History Questionnaire [4], which was completed at baseline. This summary score, available in the ABCD ~~4.0~~ Data Release, reflects maternal prenatal care, maternal substance use during pregnancy, prenatal maternal health conditions, prematurity, birth complications and developmental milestones. The scores released by the ABCD team were multiplied by (-1), so that higher positive values would indicate greater perinatal adversity. Due to data (un)availability, perinatal adversity was used as a regressor only in the non-adoptee sample.

1.5 In-Scanner Stop Signal Task (SST)

This task measures the ability to inhibit an ongoing speeded motor response to a "Go" signal [1, 5].

The ABCD version of the SST comprises two runs of 180 trials each: 150 “Go” trials, 15 “Stop” trials expected to be unsuccessful and 15 “Stop” trials expected to be successful. To maintain the breakdown of the successful/unsuccessful “Stop” trials, a tracking algorithm was implemented to alter the interval between the presentation of the ‘Go stimulus’ and the onset of the ‘Stop’ signal based on the participant’s performance. Each run was restricted to begin with a ‘Go’ trial and stop trials were separated by a minimum of one ‘Go’ trial.

Each trial lasted 1000 ms and began with the presentation of a black rightward- (50% of the time) or leftward-facing arrow (‘Go stimulus’) displayed on a mid-grey background. Participants were asked to indicate the arrow direction “as quickly and accurately as possible” using a response panel consisting of two buttons. Participants used their dominant hand to respond to the ‘Go’ stimuli and this was mapped congruently with handedness. On ‘Stop’ trials the ‘Go’ stimulus was unpredictably followed by a ‘Stop’ signal in the form of an upward facing arrow presented for 300 ms, which indicated to the participants that they should inhibit their response to the previously presented ‘Go’ signal. The presentation of the “Go” (on the “Go” trials) and “Stop” cue was followed by a fixation cross varying in duration based on participant’s reaction time for a total trial duration of 1000 ms.

1.6 MRI Data Acquisition

Scanning was performed across 21 US sites, with a protocol harmonised for Siemens Prisma, Philips, and GE 3T scanners (for details, see [5, 6]). The analyses reported here are based on the tabulated structural (sMRI) and functional magnetic resonance imaging (fMRI) data. Scanner type was controlled for in all analyses by using site id as a covariate to account for magnet and sociodemographic differences among sites. T1-weighted were acquired with an MPRAGE-PMC (Prospective Motion Correction) sequence (TR=2500 (Siemens/GE)/6.31 (Phillips) ms, TE= 2.88 (Siemens)/2.9 (Phillips)/2 (GE) ms, flip angle=8°, FOV = 256 x 256 mm, 176 (Siemens)/225 (Phillips)/208 (GE) slices of 1 × 1 mm in-plane resolution, 1 mm

thick). The fMRI data were acquired with a multiband EPI sequence (TR=800 ms, TE=30 ms, flip angle=52°, FOV = 216 x 216 mm, 60 slices of 2.4 × 2.4 mm in-plane resolution, 2.4 mm thick, multiband acceleration factor of 6).

Four resting state fMRI scans (eyes open with passive crosshair viewing), lasting 20 minutes in total, were collected in order to ensure at least 8 minutes of low-motion data. Two SST runs were also acquired for a total duration of 11:40 minutes (6).

1.7 MRI Data Preprocessing

Our analyses used tabulated sMRI and fMRI data available as part of the ABCD Study Curated Annual Release ~~4.0~~. The main processing steps applied to these data by the ABCD study team are outlined below (for further details, see [6]).

1.7.1 fMRI

Preprocessing of all functional images involved correction for head motion, spatial and gradient distortions, bias field removal, elimination of initial volumes (8 volumes [Siemens, Philips], 5 volumes [GE DV25], 16 volumes [GE DV26] to allow the MR signal to reach steady state equilibrium, normalisation of the voxel time series and co-registration of the functional images to the participant's T_1 – weighted structural image. Linear regression was used to remove from each voxel's time course quadratic trends, as well as the six motion parameters, their first derivatives, and squares (24 motion terms in total). Estimated motion time courses were filtered to attenuate signals related to respiration.

1.7.1.1 Resting-state (RS)

The following preprocessing steps were specific to the RS data (1) regression of the mean time courses of cerebral WM, ventricles, whole brain, and their first derivatives, (2) bandpass filtering of the residual time series between .009-.08 Hz; exclusion of (3) time points with framewise displacement (FD) greater than .20 mm, (4) those that were outliers in standard deviation (SD) across ROIs (i.e., SD > three times the median absolute deviation

below or above the median SD for a given participant), and of (5) time periods with fewer than 5 contiguous volumes with FD smaller than .20 mm.

Temporal variance. Based on the preprocessed data, averaged time courses were computed for cortical ROIs from an anatomically defined parcellation. Temporal variance was estimated for each ROI as an amplitude index of low frequency fluctuations, which is assumed to reflect spontaneous neural activity and is predictive of task-related responsiveness.

1.7.1.2 Task

The following preprocessing steps were specific to the task data: (1) regression of the baseline, and (2) removal of time points with FD > .90 mm. Task-specific activation strength was estimated for each individual participant using a general linear model in AFNI's 3dDeconvolve [7]. The baseline model ("null model") included regressors for average signal, quadratic trend and motion (i.e., 24 motion regressors in total, specifically, the linear and quadratic motion parameters and their derivatives). The GLM included the stimulus time series convolved with the hemodynamic response function (HRF). The latter was modelled with a gamma variate function and its temporal derivative in AFNI's SPMG option within 3dDeconvolve. Events were modelled as instantaneous. Both sets of analyses detailed below were based on the linear contrast reflecting successful inhibition (correct Stop > correct Go [baseline]).

Task-related activation. Our tests examined (1) the difference in BOLD signal between the second and the first run of the SST task, with lower values indicating decreased activation and, thus, increasing neural efficiency, potentially reflective of training effects (i.e., less activation is needed to support correct Stop performance), and (2) average BOLD signal across the two runs of the SST task, with lower values indicating overall greater neural efficiency, likely indicative of functional maturation.

Task-related variability. Our analyses focused on responses in each of the ROIs in the Destrieux anatomical atlas, specifically, (1) the difference in the standard error (SEM) of the GLM beta coefficient between the second and the first SST run, with lower values indicating greater stabilisation of the task-related response, and (2) the average SEM of the GLM beta coefficients estimated across the two SST runs, with lower SEM values indicating a more consistent response to the task-relevant information.

1.7.2 sMRI

The sMRI preprocessing pipepline included removal of non-brain tissue, corrections for gradient non-linearity distortions and intensity inhomogeneity, intensity normalisation, as well as rigid resampling and alignment to an averaged brain image in standard space. Cortical reconstruction and subcortical segmentation were performed using FreeSurfer version 5.3, where estimates of cortical thickness and volume were computed for each of the 148 ROIs from the Destrieux atlas.

1.8 Genetic Risk Scores (GRS)

MDD and AD GRSs were each computed as the weighted sum of risk alleles, as derived from the summary statistics of two large genome-wide association studies (GWASs) focused on each disorder ([8, 9] for MDD and AD, respectively), which had been made available by the original authors via the Public Results tab on the FUMA website (https://fuma.ctglab.nl/browse, 10). For AD, we computed both a composite (i.e., full) GRS and a separate Apolipoprotein E (APOE) region (chromosome 19:44.4-46.5 Mb)- vs no-APOE region GRS (cf. 11). To compute MDD and AD GRSs based on the *.genotype ABCD data, we used the PLINK genetic analysis toolset [12] with single nucleotide polymorphisms (SNPs) significant at GWAS level $p \leq 5x10^{-8}$ because these are the SNPs likely to make the most robust contribution to genetic vulnerability. However, in

supplemental analyses (see Figures S3-4), we verified that all our results are replicated when using more lenient significance thresholds for the GRS-contributing SNPs.

Prior to GRS computation, the following preprocessing steps were implemented: (1) genes with a minor allele frequency (MAF) < .05, insertion/deletion and SNPs (i.e., A/T and G/C pairs) were excluded; (2) highly correlated SNPs ($r^2 > .10$) within a 500 kb window were eliminated. The SNPs which survived the preprocessing contributed to the computation of the disorder-specific GRSs (MDD GRS: N = 8 SNPs; no-APOE AD GRS: N = 11 SNPs; APOE AD GRS = 17 SNPs; full AD GRS = 28 SNPs).

1.9 Residualisation for Confounding Variables

To minimise bias in our multivariate brain-behaviour analyses [13], only the non-imaging variables were residualised for the following confounders: sex, race (separate dummy-coded variables for "Black", "Asian", 'Mixed Race" regressed simultaneously from the non-imaging variables to account for potential differences between these racial groups and White participants), handedness, serious medical problems, scanner site, material deprivation, family conflict, neighbourhood crime, age at adoption, average modality-specific motion per participant, and chronological age (in order to estimate accelerated/decelerated neurodevelopment relative to the other participants). Due to data (un)availability, only the non-adoptee data were residualised for perinatal adversity. The adoptee and non-adoptee data were residualised separately.

1.10 MRI and GRS Data Analysis

1.10.1 Partial least squares analysis (PLS)

To provide a comprehensive description of the relationship between genetic risk for AD/MDD and neurodevelopmental timing among adoptees versus non-adoptees, we used partial least squares correlation often referred to as *PLS* [14], a multivariate technique that can identify in an unconstrained, data-driven manner, neural patterns (i.e., latent variables or

LVs) related to different conditions (i.e., task PLS) and/or individual differences variables (behavioral PLS). PLS was implemented using a series of Matlab scripts, which are available for download at https://www.rotman-baycrest.on.ca/index.php?section=345.

We thus conducted two behavioural PLS analyses featuring MDD GRS (both analyses) and either the composite AD GRS (analysis 1) or the APOE- vs no-APOE-based GRSs (analysis 2) in the “behavioural” set. In each analysis, each type of data (cortical thickness, resting state $BOLD_{SV}$, as well as $BOLD_{M}$ and $BOLD_{SV}$, averaged across the two SST runs and its difference between run 2 and run 1 of the SST) was modelled as a separate condition, whereas the adoptees and non-adoptees were modelled as separate groups. Within each group, the brain matrix contained the participants’ concatenated scores for all the data types across the 148 Destrieux ROIs. The design matrix contained a number of dummy coded variables corresponding to each condition within each group (e.g., adoptees’ cortical thickness scores). By entering both groups within the same PLS analysis we were able to identify group-specific associations between genetic vulnerability and neurodevelopmental timing.

1.10.1.1 Significance and reliability testing

In all the reported PLS analyses, the significance of each LV was determined using a permutation test (5000 permutations). In the permutation test, the rows of the brain data are randomly reordered [14]. In the case of our present analyses, PLS assigned to each ROI a weight, which reflected the contribution of the respective ROI to a specific LV. The reliability of each ROI’s contribution to a particular LV was tested by submitting all weights to a bootstrap estimation (1000 bootstraps) of the standard errors (SEs) (the bootstrap samples were obtained by sampling with replacement from the participants, [14]). In order to increase the stability of the reported results, we used a number of permutations/bootstraps greater than the standard ones (i.e., 500 permutations/100 bootstrap samples), as

recommended by [15] for use in PLS analyses of neuroimaging data. A bootstrap ratio (BSR) (weight/SE) of at least 3 in absolute value (approximate associated p-value < ~~.0001~~) was used as a threshold for identifying those ROIs that made a significant contribution to the identified LVs. The BSR is analogous to a z-score, so an absolute value greater than 2 is thought to make a reliable contribution to the LV [14], although for neuroimaging data BSR absolute values of at least 3 are recommended for use [15].

1.10.2 Mediation analyses

To test whether genetic risk for AD/MDD is linked to distinct patterns of brain and cognitive development among adoptees versus non-adoptees, we conducted three moderated mediation analyses for MDD, composite AD and no-APOE-based AD GRS using Hayes' PROCESS 3.5 macro for the Statistical Package for the Social Sciences (SPSS, [16]). PROCESS is an ordinary least squares (OLS) and logistic regression path analysis modelling tool, based on observable variables. Mediation models were tested employing 95% confidence intervals (Cis) with 50000 bootstrapping samples. In line with extant guidelines on balancing Type I and Type II errors in mediation analyses [17], the CIs for indirect effects was estimated using percentile bootstrap, which is the default option in PROCESS 3.5. As recommended [18], a heterodasticity consistent standard error and covariance matrix estimator was used. Bootstrapping-based 95% CIs for the indirect effects and for the moderation mediation index (cf. [19, 20]), as outputted by PROCESS, were used as effect size estimates.

2. Results

2.1 Regression Analyses Probing Differences in AD/MDD PRRS by Adoption Status, Sex and Race

Four linear regression analyses predicting each GRS (composite/APOE/no-APOE AD and MDD) from adoption status, race and sex (see section "Residualisation for Confounding

Variables" in the Method for coding of each variable) revealed (1) marginally greater no-APOE GRS values among adoptees relative to non-adoptees, *b* = .002, *SE* = .001, *t*(4493) = 2.018, *p* = .044; (2) greater no-APOE AD GRS values, *b* = .004, *SE* = .001, *t*(4495) = 6.538, *p* = .001, but lower MDD GRS values *b* = -.002, *SE* = .000, *t*(4495) = 13.138, *p* = .001 among Blacks (relative to Whites, Asians and mixed race individuals); (3) greater APOE, *b* = .005, *SE* = .001, *t*(4495) = 4.288, *p* = .001, and composite, *b* = .002, *SE* = .001, *t*(4495) = 3.254, *p* = .001, AD GRS values among Whites (relative to Black, Asians and mixed race individuals).

2.2 Supplementary PLS Analyses (involving only White participants)

To confirm that racial differences in genetic architecture and risk loci did not confound our investigation of AD/MDD GRS effects on neurodevelopment, we ran both PLS analyses reported in the main text using data only from White participants. The White sample encompassed 54 adoptees (30 female; 41 right-handed) and 3660 non-adoptees (1839 female; 2999 right-handed). For easier reference, the results corresponding to the full sample are depicted in Figures S1/S2-c,d, whereas Figures S1/S2-a,b feature the White-only results.

PLS 1: Neurodevelopmental patterns differentiate between genetic risk for AD vs MDD. The first PLS analysis revealed only one significant LV (*p* = .005), which accounted for 54 % of the covariance in the GRS-brain data and distinguished brain markers of genetic risk for AD from those linked to vulnerability to MDD (Figure S1-b). The brain LV was most strongly expressed in frontal, insular and inferior temporal areas (see Figure S1-a). The associated neurogenetic pattern replicated the one observed in the full sample (cf. Figure S1-c, d).

PLS 2: APOE- vs. non-APOE-based genetic vulnerability to AD is linked to distinct neurodevelopmental markers. The second PLS analysis identified a sole significant LV (*p* = .002), which accounted for 51% of the covariance in the GRS-brain data

and differentiated among brain markers of genetic risk for MDD, as well as APOE- vs non-APOE-linked vulnerability to AD (Figure S2-b). The associated brain LV was most strongly expressed in the frontal, insular and parietal areas (Figure S2-a). As in the analyses involving the full sample, the neural markers of MDD GRS tended to comprise the same functional data types as those observed in the first PLS analysis. The neurogenetic pattern associated with APOE- and non-APOE-based GRS replicated the one observed with the full sample (cf. Figure S2-c,d).

Supplemental References

References

1. Garavan, H., Bartsch, H., Conway, K., Decastro, A., Goldstein, R. Z., Heeringa, S., Jernigan, T., Potter, A., Thompson, W., & Zahs, D. (2018). Recruiting the ABCD sample: Design considerations and procedures. Developmental Cognitive Neuroscience, 32, 16–22.

2. Luciana, M., Bjork, J. M., Nagel, B. J., Barch, D. M., Gonzalez, R., Nixon, S. J., & Banich, M. T. (2018). Adolescent neurocognitive development and impacts of substance use: Overview of the adolescent brain cognitive development (ABCD) baseline neurocognition battery. Developmental Cognitive Neuroscience, 32, 67–79.

3. Barch, D. M., Albaugh, M. D., Baskin-Sommers, A., Bryant, B. E., Clark, D. B., Dick, A. S., Feczko, E., Foxe, J. J., Gee, D. G., Giedd, J., Glantz, M. D., Hudziak, J. J., Karcher, N. R., LeBlanc, K., Maddox, M., McGlade, E. C., Mulford, C., Nagel, B. J., Neigh, G., Palmer, C. E., … Xie, L. (2021). Demographic and mental health assessments in the adolescent brain and cognitive development study: Updates and age-related trajectories. Developmental Cognitive Neuroscience, 52, 101031.

4. Barch, D. M., Albaugh, M. D., Avenevoli, S., Chang, L., Clark, D. B., Glantz, M. D., Hudziak, J. J., Jernigan, T. L., Tapert, S. F., Yurgelun-Todd, D., Alia-Klein, N., Potter, A. S., Paulus, M. P., Prouty, D., Zucker, R. A., & Sher, K. J. (2018). Demographic, physical and mental health assessments in the adolescent brain and cognitive development study: Rationale and description. Developmental Cognitive Neuroscience, 32, 55–66.

5. Casey, B. J., Cannonier, T., Conley, M. I., Cohen, A. O., Barch, D. M., Heitzeg, M. M., Soules, M. E., Teslovich, T., Dellarco, D. V., Garavan, H., Orr, C. A., Wager, T. D., Banich, M. T., Speer, N. K., Sutherland, M. T., Riedel, M. C., Dick, A. S., Bjork, J. M., Thomas, K. M., Chaarani, B., … ABCD Imaging Acquisition Workgroup (2018). The Adolescent Brain

Cognitive Development (ABCD) study: Imaging acquisition across 21 sites. Developmental Cognitive Neuroscience, 32, 43–54.

6. Hagler DJ, Jr, Hatton, S., Cornejo, M.D., Makowski, C., Fair, D.A., Dick, A.S., Sutherland, M.T., Casey, B.J., Barch, D.M., Harms, M.P., Watts, R., Bjork, J.M., Garavan, H.P., Hilmer, L., Pung, C.J., Sicat, C.S., Kuperman, J., Bartsch, H., Xue, F., Heitzeg, M.M., Laird, A.R., Trinh, T.T., Gonzalez, R., Tapert, S.F., Riedel, M.C., Squeglia, L.M., Hyde, L.W., Rosenberg, M.D., Earl, E.A., Howlett, K.D., Baker, F.C., Soules, M., Diaz, J., de Leon, O.R., Thompson, W.K., Neale, M.C., Herting, M., Sowell, E.R., Alvarez, R.P., Hawes, S.W., Sanchez, W.K., Bodurka, J., Breslin, F.J., Morris, A.S., Paulus, M.P., Simmons, W.K., Polimeni, J.R., van der Kouwe, A., Nencka, A.S., Gray, K.M., Pierpaoli, C., Matochik, J.A., Noronha, A., Aklin, W.M., Conway, K., Glantz, M., Hoffman, E., Little, R., Lopez, M., Barch, V., Weiss, S.R., Wolff-Hughes, D.L., DelCarmen-Wiggins, R., Feldstein Ewing, S.W., MirandaDominguez, O., Nagel, B.J., Perrone, A.J., Sturgeon, D.T., Goldstone, A., Pfefferbaum, A., Pohl, K.M., Prouty, D., Uban, K., Bookheimer, S.Y., Dapretto, M., Galvan, A., Bagot, K., Giedd, J., Infante, M.A., Jacobus, J., Patrick, K., Shilling, P.D., Desikan, R., Li, Y., Sugrue, L., Banich, M.T., Friedman, N., Hewitt, J.K., Hopfer, C., Sakai, J., Tanabe, J., Cottler, L.B., Nixon, S.J., Chang, L., Cloak, C., Nagel, T., Reeves, G., Kennedy, D.N., Heeringa, S., Peltier, S., Schulenberg, J., Sripada, C., Zucker, R.A., Iacono, W.G., Luciana, M., Calabro, F.J., Clark, D.B., Lewis, D.A., Luna, B., Schirda, C., Brima, T., Foxe, J.J., Freedman, E.G., Mruzek, D.W., Mason, M. J., Huber, R., McGlade, E., Prescot, A., Renshaw, P.F., Yurgelun-Todd, D.A., Allgaier, N.A., Dumas, J.A., Ivanova, M., Potter, A., Florsheim, P., Larson, C., Lisdahl, K., Charness, M.E., Fuemmeler, B., Hettema, J.M., Maes, H.H., Florsheim, J., Anokhin, A.P., Glaser, P., Heath, A.C., Madden, P.A., Baskin-Sommers, A., Constable, R.T., Grant, S.J., Dowling, G.J., Brown, S.A., Jernigan, T.L., Dale, A.M.,

2019. Image processing and analysis methods for the Adolescent Brain Cognitive Development Study. NeuroImage, 202, 116091.

7. Cox, R.W. (1996) AFNI: software for analysis and visualization of functional magnetic resonance neuroimages. *Computers and Biomedical Research, 29*, 162-173.

8. Howard, D. M., Adams, M. J., Clarke, T. K., Hafferty, J. D., Gibson, J., Shirali, M. et al. (2019). Genome-wide meta-analysis of depression identifies 102 independent variants and highlights the importance of the prefrontal brain regions. Nature neuroscience, 22, 343–352.

9. Kunkle, B. W., Grenier-Boley, B., Sims, R., Bis, J. C., Damotte, V., Naj, A. C et al. (2019). Genetic meta-analysis of diagnosed Alzheimer's disease identifies new risk loci and implicates Aβ, tau, immunity and lipid processing. Nature genetics, 51, 414–430.

10. Watanabe, K., Taskesen, E., van Bochoven, A., & Posthuma, D. (2017). Functional mapping and annotation of genetic associations with FUMA. Nature Communications, 8, 1826.

11. Leonenko, G., Shoai, M., Bellou, E., Sims, R., Williams, J., Hardy, J., Escott-Price, V., & Alzheimer's Disease Neuroimaging Initiative (2019). Genetic risk for Alzheimer Disease is distinct from genetic risk for amyloid deposition. Annals of Neurology, 86, 427–435.

12. Chang, C. C., Chow, C. C., Tellier, L. C., Vattikuti, S., Purcell, S. M., & Lee, J. J. (2015). Second-generation PLINK: rising to the challenge of larger and richer datasets. GigaScience, 4, 7.

13. Winkler, A. M., Renaud, O., Smith, S. M., & Nichols, T. E. (2020). Permutation inference for canonical correlation analysis. NeuroImage, 220, 117065.

14. Krishnan, A., Williams, L. J., McIntosh, A. R., & Abdi, H. (2011). Partial Least Squares (PLS) methods for neuroimaging: a tutorial and review. NeuroImage, 56, 455–475.

15. McIntosh, A. R., & Lobaugh, N. J. (2004). Partial least squares analysis of neuroimaging data: applications and advances. NeuroImage, 23 Suppl 1, S250–S263.

16. Hayes, A.F. (2018) Introduction to Mediation, Moderation, and Conditional Process Analysis: A Regression-Based Approach. (Second Edition) Guilford Press: New York.

17. Hayes, A.F. & Scharkow, M. (2013) The relative trustworthiness of inferential tests of the indirect effect in statistical mediation analysis: does method really matter? Psychological Science, 10, 1918-1927.

18. Hayes, A. & Cai, L. (2007) Using heteroskedasticity-consistent standard error estimators in OLS regression: An introduction and software implementation. Behavior Research Methods, 39, 709-722.

19. Miočević, M., O'Rourke, H. P., MacKinnon, D. P., & Brown, H. C. (2018). Statistical properties of four effect-size measures for mediation models. *Behavior Research Methods*, 50, 285–301.

20. Walters, G.D. (2018) PM effect size estimation for mediation analysis: a cautionary note, alternate strategy, and real data illustration. *International Journal of Social Research Methodology, 21*, 25-33

Table S1
SNPs Contributing to the AD and MDD GRSs

GRS	SNP	Nearest Gene
APOE AD	rs55848260	CEACAM22P
	rs7260482	CTB-171A8.1
	rs2965101	snoZ6
	rs2927437	BCL3
	rs4803750	BCL3
	rs10408993	CBLC
	rs28399637	BCAM
	rs10409208	PVRL2
	rs73572039	PVRL2
	rs6859	PVRL2
	rs157580	TOMM40
	rs2075650	TOMM40
	rs16979595	CLPTM1
	rs3786507	CLASRP
	rs10405086	MARK4:PPP1R37
	rs10415983	MARK4:AC006126.3
	rs11083767	MARK4:AC006126.3
No-APOE AD	rs6701713	CR1
	rs13025765	BIN1
	rs7561528	BIN1
	r6931478	CD2AP
	rs11767557	EPHA1-AS1

	rs11136000	CLU
	rs11039149	NR1H3
	rs935914	SPI1
	rs4938933	MS4A4A
	rs3851179	RNU6-560P
	rs3764650	ABCA7
MDD	rs12127789	NEGR1
	rs2568958	RPL31P12
	rs72710803	RP1-35C21.2
	rs200949	HIST1H1B
	rs997934	ADARB2
	rs867626	SORCS3
	rs2187490	Y_RNA
	rs55943003	CTD-2171N6.1

Note. SNP = single nucleotide polymorphism. GRS = genetic risk score. AD = Alzheimer's Disease. MDD = Major Depressive Disorder.

Genetic Risk—Structural (Cortical Thickness) and Functional (Inhibitory Control) Neurodevelopment (Composite AD GRS)

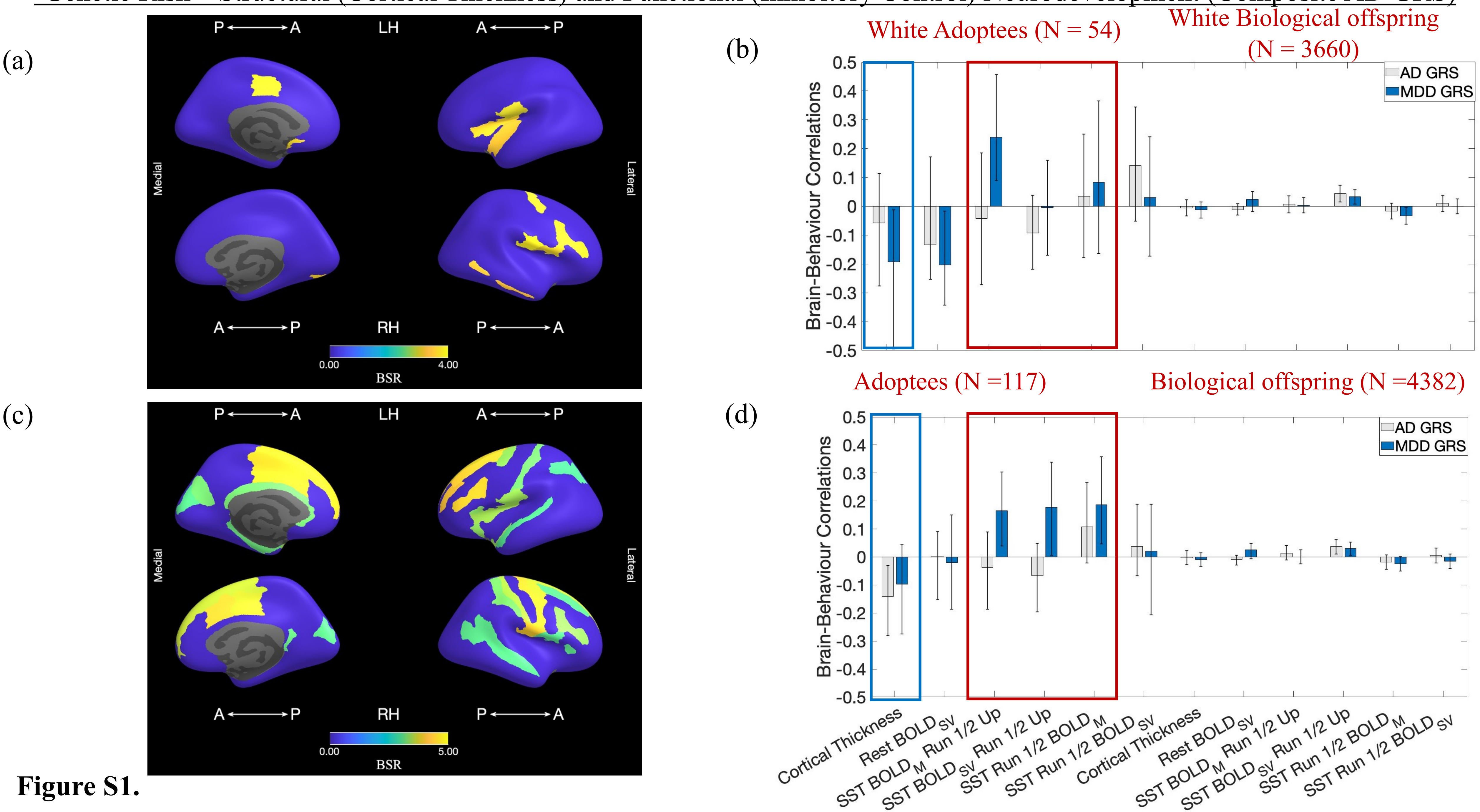

Figure S1.

Genetic Risk—Structural (Cortical Thickness) and Functional (Inhibitory Control) Neurodevelopment (APOE- vs. no-APOE-based AD GRS)

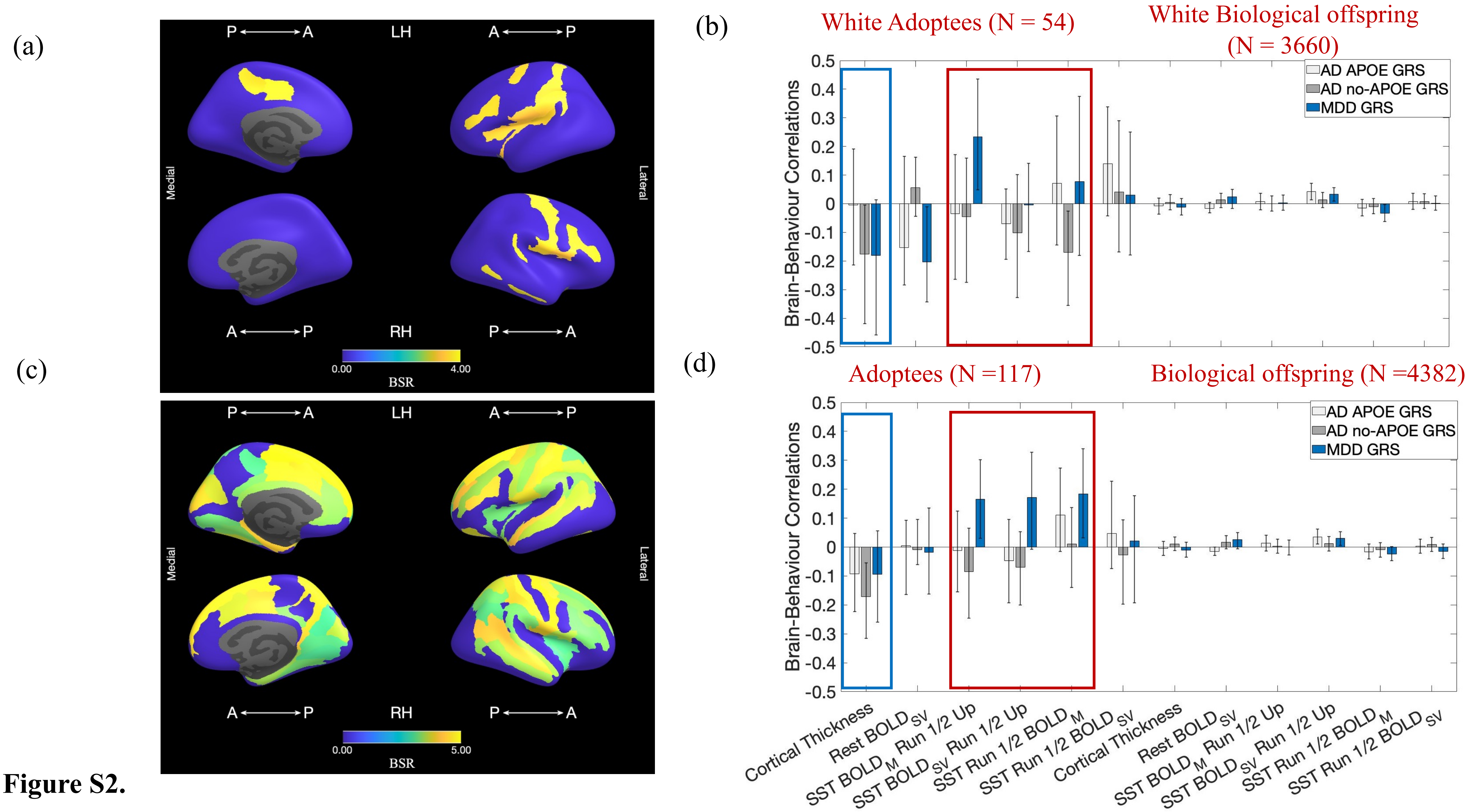

Figure S2.

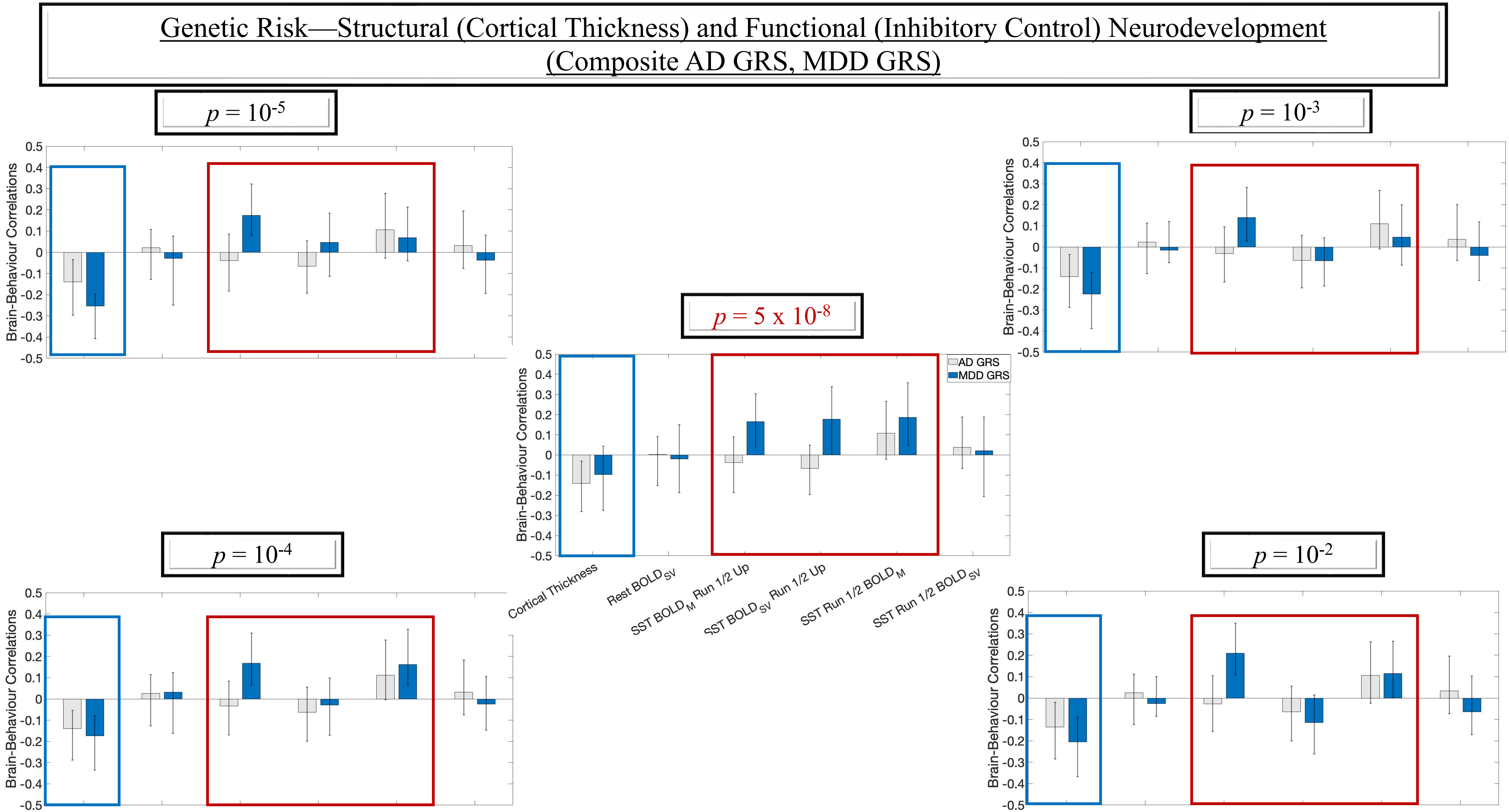
Genetic Risk—Structural (Cortical Thickness) and Functional (Inhibitory Control) Neurodevelopment
(Composite AD GRS, MDD GRS)
p = 10-5
p = 10-3
p = 5 x 10-8
p = 10-4
p = 10-2
AD GRS
MDD GRS
Brain-Behaviour Correlations
Cortical Thickness
Rest BOLD SV
SST BOLD M Run 1/2 Up
SST BOLD SV Run 1/2 Up
SST Run 1/2 BOLD M
SST Run 1/2 BOLD SV

Figure S3.

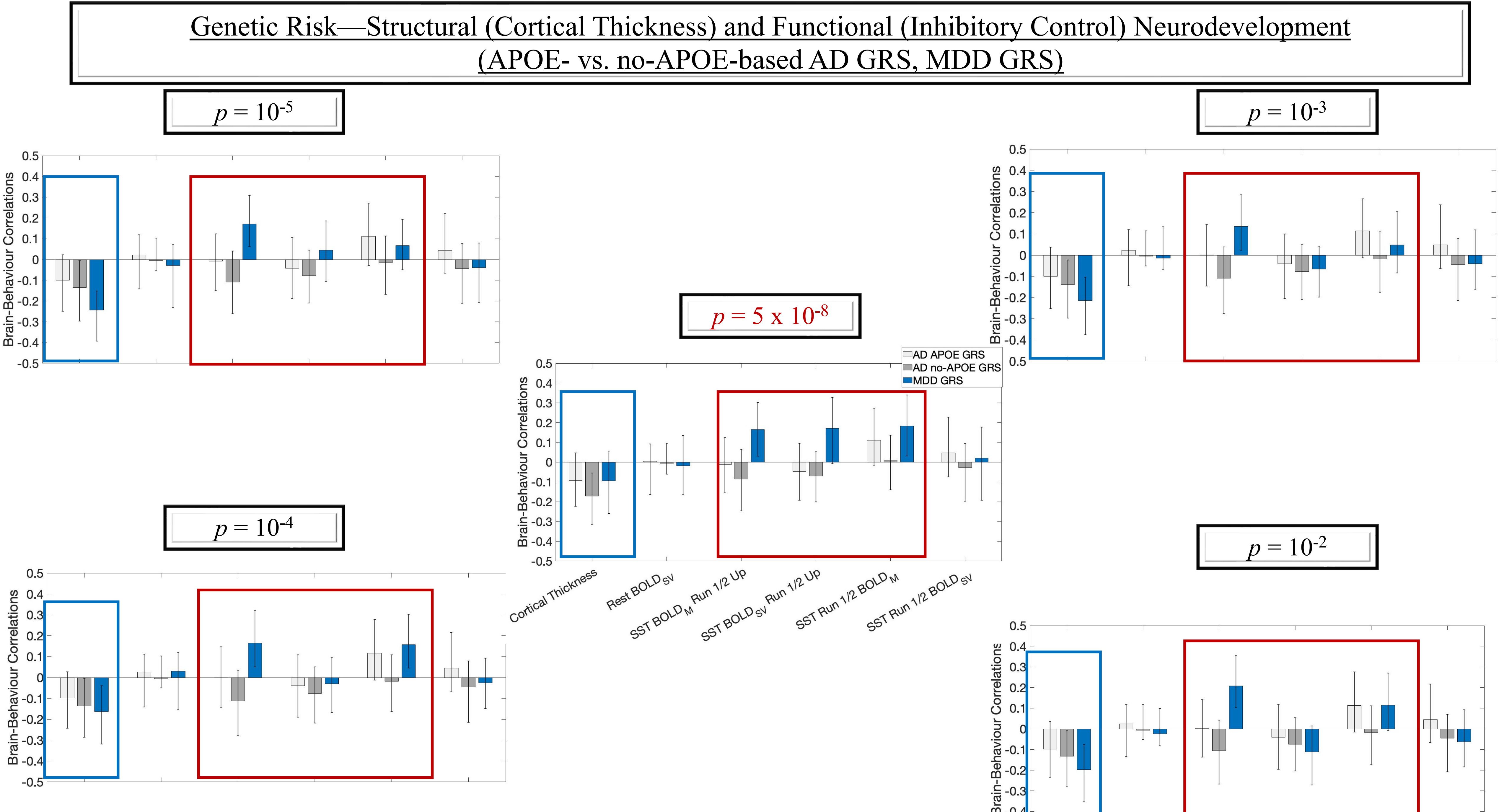
Genetic Risk—Structural (Cortical Thickness) and Functional (Inhibitory Control) Neurodevelopment
(APOE- vs. no-APOE-based AD GRS, MDD GRS)
$p = 10^{-5}$
$p = 10^{-3}$
$p = 5 \times 10^{-8}$
$p = 10^{-4}$
$p = 10^{-2}$
AD APOE GRS
AD no-APOE GRS
MDD GRS
Brain-Behaviour Correlations
Cortical Thickness
Rest $BOLD_{SV}$
SST $BOLD_M$ Run 1/2 Up
SST $BOLD_{SV}$ Run 1/2 Up
SST Run 1/2 $BOLD_M$
SST Run 1/2 $BOLD_{SV}$

Figure S4.

Supplemental Figure Captions

Figure S1. Results of the supplemental behavioural-PLS analysis 1 linking neurodevelopment to MDD and the composite AD GRSs. Panels (b) and (d) show the correlations between the LV brain scores and the GRSs. Error bars are the 95% CIs from the bootstrap procedure. CIs that do not include zero reflect robust correlations between the respective GRS and the brain score in a given condition (i.e., data type) across all participants. Panels (a) and (c) depict the Destrieux ROIs with robust loadings on the LVs in panels (b) and (d), respectively, and visualized with the Freesurfer Surface (https://chrisadamsonmcri.github.io/freesurfer_statsurf_display). In the brain figures in panels (a) and (c), absolute BSR values lower than 3 have been set to zero. Rest $BOLD_{SV}$ = amplitude index of resting state low frequency fluctuations in BOLD signal. SST $BOLD_{M}$ Run 1/2 Up = difference between the GLM-derived run 2 and run 1 betas, based on the Correct Stop > Correct Go contrast. SST $BOLD_{SV}$ Run 1/2 Up = difference between the GLM-derived SEMs associated with the run 2 and run 1 beta, respectively, based on the Correct Stop > Correct Go contrast. SST Run 1/2 $BOLD_{M}$ = average of the GLM-derived run 2 and run 1 betas, based on the Correct Stop > Correct Go contrast. SST Run 1/2 $BOLD_{SV}$ = average of the GLM-derived SEMs associated with the run 2 and run 1 beta, respectively, based on the Correct Stop > Correct Go contrast. LV = latent variable. AD = Alzheimer's Disease. MDD = Major Depressive Disorder. GRS = genetic risk score. BSR = bootstrap ratio. CI = confidence interval. SST = Stop-Signal Task. LH = left hemisphere. RH = right hemisphere. GLM = general linear model.

Figure S2. Results of the supplemental behavioural-PLS analysis 2 linking neurodevelopment to MDD and APOE vs no-APOE AD GRSs. Panels (b) and (d) show the correlations between the LV brain scores and the GRSs. Error bars are the 95% CIs from the bootstrap procedure. CIs that do not include zero reflect robust correlations between the respective GRS and the

brain score in a given condition (i.e., data type) across all participants. Panels (a) and (c) depict the Destrieux ROIs with robust loadings on the LVs in panels (b) and (d), respectively, and visualized with the Freesurfer Surface (https://chrisadamsonmcri.github.io/freesurfer_statsurf_display). In the brain figures in panels (a) and (c), absolute BSR values lower than 3 have been set to zero. Rest $BOLD_{SV}$ = amplitude index of resting state low frequency fluctuations in BOLD signal. SST $BOLD_{M}$ Run 1/2 Up = difference between the GLM-derived run 2 and run 1 betas, based on the Correct Stop > Correct Go contrast. SST $BOLD_{SV}$ Run 1/2 Up = difference between the GLM-derived SEMs associated with the run 2 and run 1 beta, respectively, based on the Correct Stop > Correct Go contrast. SST Run 1/2 $BOLD_{M}$ = average of the GLM-derived run 2 and run 1 betas, based on the Correct Stop > Correct Go contrast. SST Run 1/2 $BOLD_{SV}$ = average of the GLM-derived SEMs associated with the run 2 and run 1 beta, respectively, based on the Correct Stop > Correct Go contrast. LV = latent variable. AD = Alzheimer's Disease. MDD = Major Depressive Disorder. GRS = genetic risk score. BSR = bootstrap ratio. CI = confidence interval. SST = Stop-Signal Task. LH = left hemisphere. RH = right hemisphere. GLM = general linear model.

Figure S3. Replication of the LV1-linked pattern (PLS analysis 1, see Figure 2-b), using a variety of *p*-thresholds (10^{-5}, 10^{-4}, 10^{-3}, 10^{-2}) for selecting the composite AD/MDD GRS-contributing variants. For easier visualisation, the PLS conditions are represented only in the adoptee group in which significant brain-GRS associations had been detected with a stringent p-value threshold (5×10^{-8}). LV= latent variable. GRS = genetic risk score. AD = Alzheimer's Disease. MDD = Major Depressive Disorder. SST = Stop-Signal Task. Rest $BOLD_{SV}$ = amplitude index of resting state low frequency fluctuations in BOLD signal. SST $BOLD_{M}$ Run 1/2 Up = difference between the GLM-derived run 2 and run 1 betas, based on the Correct Stop > Correct Go contrast. SST $BOLD_{SV}$ Run 1/2 Up = difference between the

GLM-derived SEMs associated with the run 2 and run 1 beta, respectively, based on the Correct Stop > Correct Go contrast. SST Run 1/2 $BOLD_{M}$ = average of the GLM-derived run 2 and run 1 betas, based on the Correct Stop > Correct Go contrast. SST Run 1/2 $BOLD_{SV}$ = average of the GLM-derived SEMs associated with the run 2 and run 1 beta, respectively, based on the Correct Stop > Correct Go contrast. GLM = general linear model.

Figure S4. Replication of the LV1-linked pattern (PLS analysis 2, see Figure 2-d), using a variety of *p*-thresholds (10^{-5}, 10^{-4}, 10^{-3}, 10^{-2}) for selecting the APOE/no-APOE AD/MDD GRS-contributing variants. For easier visualisation, the PLS conditions are represented only in the adoptee group in which significant brain-GRS associations had been detected with a stringent p-value threshold (5×10^{-8}). LV= latent variable. GRS = genetic risk score. AD = Alzheimer's Disease. MDD = Major Depressive Disorder. SST = Stop-Signal Task. Rest $BOLD_{SV}$ = amplitude index of resting state low frequency fluctuations in BOLD signal. SST $BOLD_{M}$ Run 1/2 Up = difference between the GLM-derived run 2 and run 1 betas, based on the Correct Stop > Correct Go contrast. SST $BOLD_{SV}$ Run 1/2 Up = difference between the GLM-derived SEMs associated with the run 2 and run 1 beta, respectively, based on the Correct Stop > Correct Go contrast. SST Run 1/2 $BOLD_{M}$ = average of the GLM-derived run 2 and run 1 betas, based on the Correct Stop > Correct Go contrast. SST Run 1/2 $BOLD_{SV}$ = average of the GLM-derived SEMs associated with the run 2 and run 1 beta, respectively, based on the Correct Stop > Correct Go contrast. GLM = general linear model.
